# Regulation of Heterogenous LexA Expression in *Staphylococcus aureus* by an Antisense RNA Originating from Transcriptional Read-Through upon Natural Mispairings in the *sbrB* Intrinsic Terminator

**DOI:** 10.3390/ijms23010576

**Published:** 2022-01-05

**Authors:** Laurène Bastet, Pilar Bustos-Sanmamed, Arancha Catalan-Moreno, Carlos J. Caballero, Sergio Cuesta, Leticia Matilla-Cuenca, Maite Villanueva, Jaione Valle, Iñigo Lasa, Alejandro Toledo-Arana

**Affiliations:** 1 Instituto de Agrobiotecnología (IdAB), Consejo Superior de Investigaciones Científicas (CSIC)-Gobierno de Navarra, Avda. de Pamplona 123, 31192 Mutilva, Spain; laurene.bastet@unavarra.es (L.B.); pilar.bustos@csic.es (P.B.-S.); arancha.catalan@csic.es (A.C.-M.); carlos.caballero@unavarra.es (C.J.C.); sergio.cuesta@csic.es (S.C.); leticia.matilla@unavarra.es (L.M.-C.); maite.villanueva@unavarra.es (M.V.); jaione.valle@csic.es (J.V.); 2 Laboratory of Microbial Pathogenesis, Navarrabiomed-Universidad Pública de Navarra (UPNA)-Hospital Universitario de Navarra-IdiSNA, 31008 Pamplona, Spain; ilasa@unavarra.es

**Keywords:** antisense RNA, transcriptional termination, transcriptional read-through, *lexA*, post-transcriptional regulation, *Staphylococcus aureus*

## Abstract

Bacterial genomes are pervasively transcribed, generating a wide variety of antisense RNAs (asRNAs). Many of them originate from transcriptional read-through events (TREs) during the transcription termination process. Previous transcriptome analyses revealed that the *lexA* gene from *Staphylococcus aureus*, which encodes the main SOS response regulator, is affected by the presence of an asRNA. Here, we show that the *lexA* antisense RNA (*lexA*-asRNA) is generated by a TRE on the intrinsic terminator (TT*_sbrB_*) of the *sbrB* gene, which is located downstream of *lexA*, in the opposite strand. Transcriptional read-through occurs by a natural mutation that destabilizes the TT*_sbrB_* structure and modifies the efficiency of the intrinsic terminator. Restoring the mispairing mutation in the hairpin of TT*_sbrB_* prevented *lexA*-asRNA transcription. The level of *lexA*-asRNA directly correlated with cellular stress since the expressions of *sbrB* and *lexA*-asRNA depend on the stress transcription factor SigB. Comparative analyses revealed strain-specific nucleotide polymorphisms within TT*_sbrB_*, suggesting that this TT could be prone to accumulating natural mutations. A genome-wide analysis of TREs suggested that mispairings in TT hairpins might provide wider transcriptional connections with downstream genes and, ultimately, transcriptomic variability among *S. aureus* strains.

## 1. Introduction

The discovery of pervasive transcription was an unprecedented phenomenon that changed the way we understand gene regulation. Thanks to the new RNA sequencing technologies, thousands of new non-coding RNAs (ncRNAs) were uncovered in almost every sequenced organism, highlighting the importance of post-transcriptional regulation [1]. Transcriptome mapping analyses in bacterial models such as *Escherichia coli*, *Vibrio cholera*, *Helicobacter pylori* and *Staphylococcus aureus* show a high rate of antisense RNAs (asRNAs) that overlap with ~40 to 75% of the coding sequences (CDSs) [2,3,4,5,6]. In general, the length of asRNAs ranges from just a few nucleotides to over a thousand. While some of the antisense transcripts pair with a specific region of the messenger RNA (mRNA), others can overlap with entire genes or operons [7,8]. These long asRNAs often originate from transcriptional read-through events (TREs) of transcriptional terminators (TTs) located in the intergenic regions (IGRs) of convergent CDSs [9,10,11,12,13,14,15,16].

Transcription termination in bacteria is controlled by two coexisting mechanisms. The first mechanism consists in the formation of hairpin structures followed by U-rich stretches in nascent RNA molecules, which promote the RNA polymerase (RNAP) dissociation from the DNA template. Intrinsic termination does not require additional factors for termination to occur [17]. However, a subclass of weak non-canonical terminators, including weak hairpins and/or distal U-tract interruptions, need the transcription elongation factor NusA for effective termination [18]. Deletion of NusA produces transcriptional read-through of NusA-dependent terminators that causes the misregulation of genes involved in essential cellular functions [18].

In the second mechanism, transcription termination requires the interaction of the Rho factor, an ATP-dependent helicase/translocase that scans the nascent RNA for termination signals to promote RNAP dissociation. The traditional model of Rho-dependent transcriptional termination postulates that Rho loads itself onto the nascent RNA and translocates through the molecule in search of the active RNAP to pull the RNA from it, arresting transcription [17]. However, this model is incompatible with recent structural, biochemical and genetic data. The revised model proposes that prior to interacting with the nascent transcript, Rho binds the RNAP, NusA and NusG complex. The RNA exiting the RNAP interacts with NusA before entering the Rho channel, which scans for termination signals (*rut*). Once a *rut* signal emerges from RNAP, Rho rearranges its conformation to capture it. This conformational change leads to irreversible inhibition of the elongation complex that terminates RNA transcription [19,20,21]. Interestingly, inhibition of Rho activity increases pervasive transcription both in Gram-positive and Gram-negative bacteria [13,15,22,23,24].

Overlapping antisense transcripts could be originated from convergent genes encoded in opposite DNA strands due to the presence of leaky intrinsic terminators or depletion of Rho activity. Although the physiological consequences of antisense transcription are still debated, it seems that it could contribute to phenotypic variations (by creating fluctuations on gene expression) during bacterial growth and allow adaptation and survival in the ever-changing environmental conditions [8,15,16,25,26].

In this study, we used *S. aureus* as a model to understand the impact of TREs in transcription regulation. Specifically, we focused on the asRNA of the *lexA* gene (*lexA*-asRNA) [4]. LexA is the master regulator of the bacterial SOS response and it behaves as a transcriptional repressor of the SOS genes involved in DNA reparation, mutagenesis and cell cycle arrest [27]. When the DNA is damaged, LexA is inactivated and, hence, the expression of the SOS genes is promoted. Our RNA-Seq analysis of the *S. aureus* 15981 strain indicated that the *lexA*-asRNA originates from the transcription of the neighboring gene, *sbrB* [4]. The *sbrB* gene encodes SbpB, a small basic protein of unknown function. Its expression is activated by the alternative sigma factor B (SigB), which modulates the stress response of several Gram-positive bacteria [28,29]. The expression of *lexA*-asRNA depends on the read-through of the *sbrB* intrinsic terminator (TT*_sbrB_*). We demonstrated that such read-through occurs thanks to a natural mutation within TT*_sbrB_* that causes a hairpin mispairing. Conservation analyses among thousands of *S. aureus* genomes unveiled mispairings within the TT*_sbrB_* sequence resulting in different *lexA*-asRNA levels. Our study also suggests that mispairings in TTs (either evolutionarily fixed or temporarily acquired by mutations) might be a widespread phenomenon in *S. aureus* genomes. This could provide an unexplored source of variability by transcriptionally connecting contiguous genes.

## 2. Results

### 2.1. The lexA Antisense RNA Originates from a Transcriptional Read-Through Event of an Upstream Terminator

**Figure 1:**
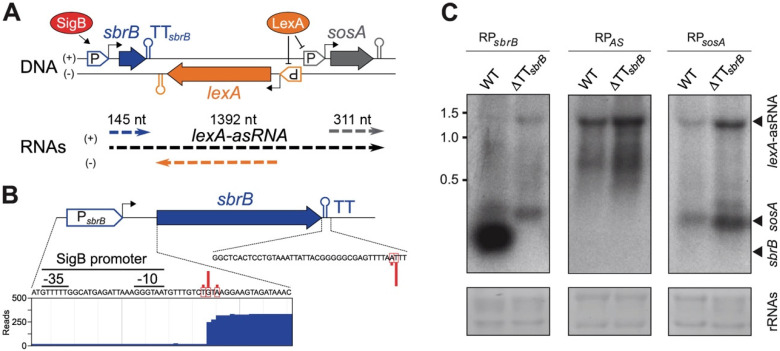
*lexA*-asRNA is produced from the SigB-dependent *sbrB* promoter upon transcriptional read-through of TT*_sbrB_*. (**A**) Schematic representation of the *sbrB-lexA-sosA* locus according previous transcriptomic maps [4]. ORFs and promoters (P) from *sbrB*, *lexA* and *sosA* genes are represented as blue, orange and gray arrows, respectively. Transcriptional terminators (TT) are illustrated as colored hairpins. Transcripts (RNAs) generated from both DNA strands are represented as dashed arrows. (**B**) Validation of *sbrB* mRNA boundaries. The transcriptional start and termination sites of the *sbrB* were determined by visualizing the *S. aureus* TSS sequencing data [30] and performing a simultaneous mapping of the 5′ and 3′ mRNA ends by circularization (mRACE) [31]. A Jbrowser image showing the RNA-Seq reads mapping on the *sbrB* promoter region is also included. The complete transcriptomic map is available at http://rnamaps.unavarra.es, accessed on 8 December 2021 [7]. Red bars represent the frequency of each nucleotide position at the 5′ and 3′ ends identified by mRACE. (**C**) Northern blots showing the *sbrB*, *lexA*-asRNA and *sosA* mRNA levels expressed from the *S. aureus* WT and ΔTT*sbrB* strains. Transcripts were developed using ^32^P-radiolabelled riboprobes designed to specifically target the *sbrB* (RP*_sbrB_*), *lexA*-asRNA (RP*_AS_*) and *sosA* (RP*_sosA_*) mRNAs. The single-stranded transcript sizes from the RNA Millennium marker are indicated. Midori green-stained ribosomal RNAs are included as loading controls.

Our previous transcriptome mapping revealed the presence of an antisense transcript that overlapped with the *lexA* gene in *S. aureus* 15981, a clinical isolate (Figure 1A) [4]. We hypothesized that *lexA*-asRNA was originated from the SigB dependent-promoter of the *sbrB* gene, which encodes SbpB, a small protein of 38 amino acids [28]. To validate this hypothesis, we first confirmed the transcriptional start and termination sites (TSS and TTS, respectively) of the *sbrB* mRNA by visualizing *S. aureus* TSS sequencing data [30] and performing simultaneous mapping of the 5′ and 3′ mRNA ends by circularization (mRACE) [31]. Figure 1B shows that transcription initiates 9 bp downstream of the SigB-promoter sequence (P*_sbrB_*), GTTT-N_16_-GGGTAA (-35/-10), identified by Nielsen et al. [28], while ending 145 nt away from the TSS (Figure 1B). We then constructed a chromosomal mutant by deleting the TT*_sbrB_* from the *sbrB* gene in the *S. aureus* 15981 strain. The wild-type (WT) and mutant strains were grown until the mid-log phase and total RNA was extracted. Northern blots were performed using three strand-specific riboprobes (RP) that targeted the antisense region of the *lexA* (RP_AS_), *sbrB* (RP*_sbrB_*) and *sosA* (RP*_sosA_*) mRNAs (Figure 1A). In agreement with our previous study [4], RP_AS_ revealed a transcript of approximately 1.4 kb in the WT strain (Figure 1C). The expression of such transcript was considerably higher in the ΔTT*_sbrB_* strain than in WT strain (Figure 1C). Similar *lexA*-asRNA expression patterns were obtained when using the RP*_sbrB_*- and RP*_sosA_*-specific riboprobes. As expected, RP*_sbrB_* revealed an additional ∼150 nt mRNA band corresponding to the *sbrB* transcript in the WT strain. However, this small transcript was absent in the ΔTT*_sbrB_* mutant, which lacked the transcriptional termination signal downstream of the *sbrB* gene (Figure 1C). When using RP*_sosA_*, a ∼300 nt band corresponding to the *sosA* mRNA was observed for both strains. Such transcript (*sosA* mRNA) originated from the P*_sosA_* promoter (Figure 1A,C). These results indicate that the levels of *lexA*-asRNA are driven by the rate of transcriptional read-through at TT*_sbrB_*. When read-through occurs, *lexA*-asRNA becomes a polycistronic operon that comprises the *sbrB* and *sosA* CDSs, as well as the long non-coding region that overlaps with the *lexA* mRNA. This transcriptional architecture resembles that of a non-contiguous operon organization [7].

### 2.2. Alkaline Stress Increases lexA-asRNA Expression through SigB Activation

**Figure 2:**
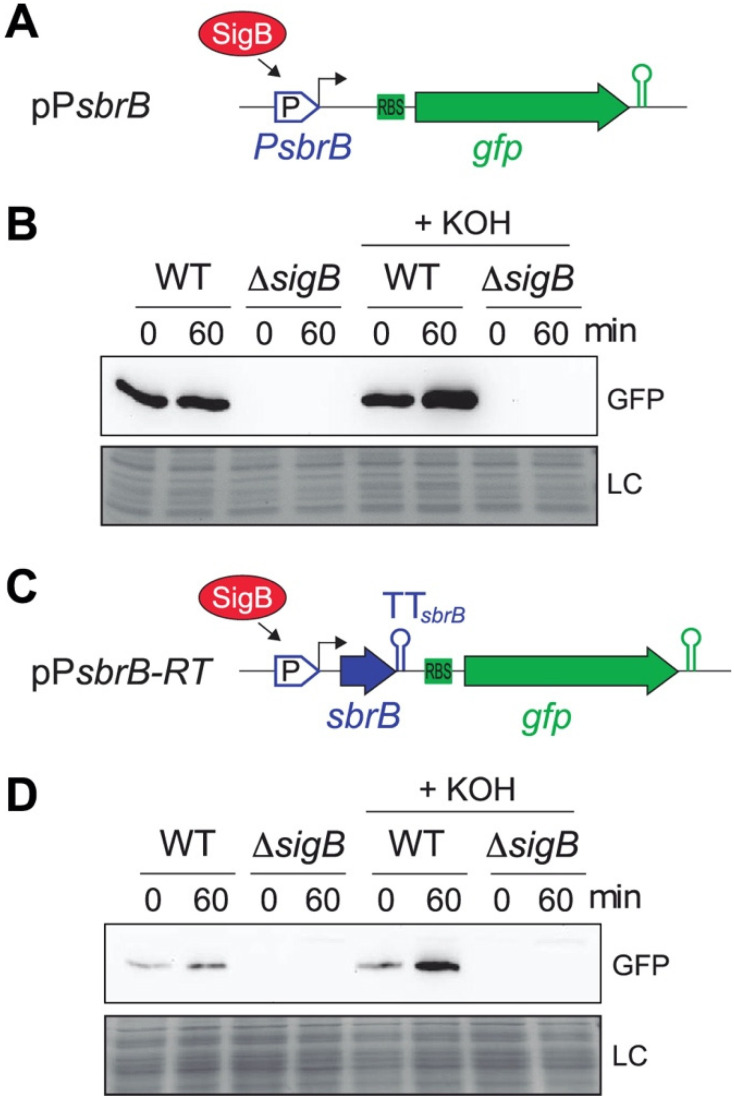
Alkaline stress activates the *sbrB* promoter and *lexA*-asRNA expression. Schematic representations of plasmid constructions including (**A**) the P*_sbrB_* transcriptional reporter and (**C**) TT*_sbrB_* transcriptional read-through reporter. (**B**,**D**) Western blots showing the GFP protein levels from the WT and Δ*sigB* strain transformed by plasmids illustrated in A and C, respectively. Bacteria were grown until exponential phase and, when necessary, challenged with KOH for 60 min to induce alkaline stress. Proteins were transferred to nitrocellulose membranes, incubated with anti-GFP monoclonal antibodies and developed using peroxidase-conjugated goat anti-mouse antibodies and a bioluminescence kit. Coomassie stain gel portions are included as loading controls.

Nielsen et al. reported that alkaline stress by exposure to KOH activates SbrB expression in *S. aureus* [28]. Since *lexA*-asRNA appears to be under the control of the P*_sbrB_* promoter, we hypothesized that KOH addition would increase its levels. To confirm this, we fused the P*_sbrB_* promoter to the green fluorescent protein (*gfp*) reporter gene, generating the pP*_sbrB_* plasmid (Figure 2A). *S. aureus* 15981 WT and its isogenic *sigB* mutant (Δ*sigB*) strains carrying the pP*_sbrB_* reporter were grown until mid-log phase in MH media at 37 °C and challenged with 30 mM of KOH. Total protein samples were collected before and after 1 h of KOH addition and their GFP levels monitored by Western blot. The results showed an increase in GFP expression when the WT strain was incubated with KOH. In contrast, no GFP was detected in the Δ*sigB* mutant strain regardless of the KOH treatment (Figure 2B), confirming that the alkaline stress activates P*_sbrB_* in a SigB-dependent manner. Next, to monitor the read-through levels of TT*_sbrB_*, we constructed a plasmid carrying the *gfp* reporter gene located downstream of TT*_sbrB_* (pP*_sbrB_* RT) (Figure 2C). Western blot analyses revealed that the expression of GFP was induced in the WT strain upon exposition to alkaline stress, whereas no bands were detected in the Δ*sigB* mutant (Figure 2D). Altogether, these results strongly indicate that the *lexA*-asRNA transcript levels depend on SigB activity.

### 2.3. Translation of SbpB Does Not Affect the Transcriptional Read-Through of TT_sbrB_

**Figure 3:**
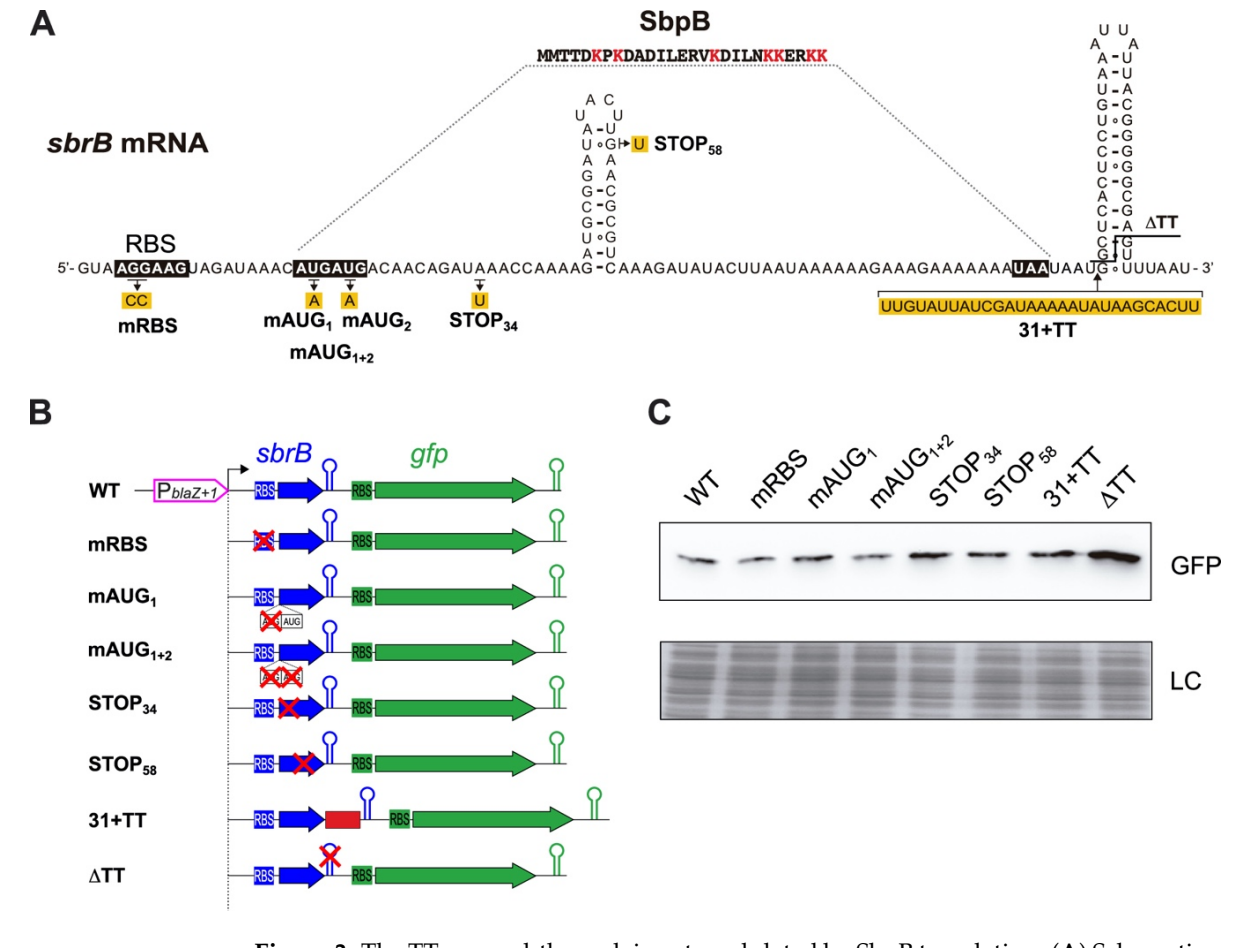
The TT*_sbrB_* read-through is not modulated by SbpB translation. (**A**) Schematic representation of the *sbrB* mRNA indicating the ribosome binding site (RBS), the putative start codons (AUG), the small protein SbpB sequence (lysines are colored in red) and the transcriptional terminator structure. The nucleotide modifications performed in the *sbrB* mRNA are highlighted in yellow. (**B**) Schematic representation of the different plasmids harboring translation and the transcriptional read-through reporters, respectively. WT and mutant mRNAs were expressed under the control of the P*_blaZ+_*_1_ promoter. (**C**) Western blot analyses showing the GFP levels produced from the different plasmids. Membranes were incubated with monoclonal anti-GFP and developed with peroxidase-conjugated goat anti-mouse antibodies and a bioluminescence kit. Coomassie gel portions are included as loading controls (LC).

After confirming the transcriptional control exerted by SigB over *lexA*-asRNA, we wondered whether the transcriptional read-through of TT*_sbrB_* could be actively modulated. Upon closer inspection of the SbpB CDS, we noticed that the SbpB CDS contained several lysine codons (four concentrated in the last six amino acids of the C-terminus) (Figure 3A), which could theoretically act as a transcription attenuation mechanism, as previously described [32,33,34]. Antisense RNAs transcription controlled by riboswitches had already been characterized in *Clostridium acetobutylicum* and *Listeria monocytogenes* [35,36]. Since this hypothesis relied on the translation of SbpB, we tested whether mutations in the *sbrB* mRNA that affect translation would also alter transcription termination. We generated a plasmid carrying a transcriptional fusion of the *gfp* gene downstream of TT*_sbrB_* and under the control of the P*_blaZ_*_+1_ promoter, which is constitutively expressed. Then, we mutated key translational elements of the *sbrB* mRNA such as the RBS and both AUG putative start codons (SbpB has two possible consecutive start codons) and introduced premature STOP codons in the SbpB CDS (pRT plasmid series, Table S2 and Figure 3B). The expected effects of these mutations on the translation of *sbrB* were controlled by constructing orthologous translational GFP reporter plasmids and monitoring the expression of the SbpB–GFP chimera by Western blots (pTL plasmid series, Table S2 and Figure S1). Additionally, we designed a construct that introduced a separation module of 31 nucleotides in order to generate a gap between the stop codon and TT*_sbrB_* (pRT 31+TT). Finally, we included a version in which TT*_sbrB_* was deleted (pRT ΔTT, Figure 3B). Western blots showed that despite an increase in the GFP intensity when TT*_sbrB_* was deleted, none of the mutation variants were critical enough to produce significant expression changes (Figure 3C). These results suggest that the translation of SbpB has no effect on the transcription termination of its mRNA, at least in the tested conditions.

### 2.4. A Single Nucleotide Change (G112A) in TT_sbrB_ of S. aureus Is Responsible for Its Transcriptional Read-Through

**Figure 4:**
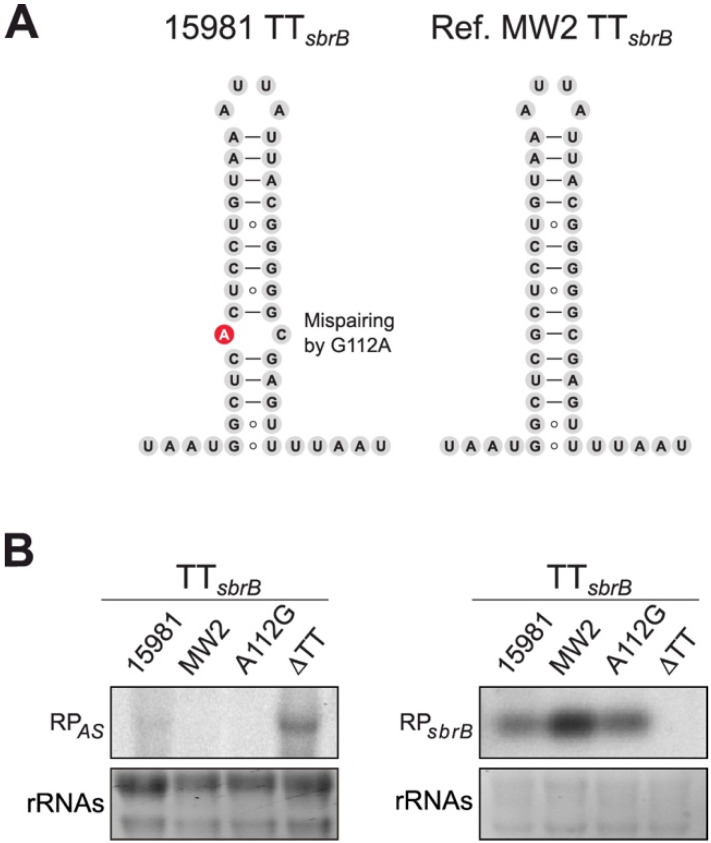
A single nucleotide change is responsible for the TT*_sbrB_* read-through. (**A**) Putative TT*_sbrB_* structures of the *S. aureus* 15981 and MW2 strains. The mutation found in the *S. aureus* 15981 strain that generated the nucleotide mispairing in the TT*_sbrB_* is highlighted in red. (**B**) Northern blot analyses showing the *lexA*-asRNA and *sbrB* expression levels from the *S. aureus* 15981, MW2, A112G and ΔTT strains. Bacteria were grown at 37 °C in MHg until exponential phase (OD_600_ 0.3). Midori green-stained ribosomal RNAs are included as loading controls.

Previous studies have shown that transcription termination depends on the sequence specificity and secondary structure of the terminator stem-loop. Variations in the stem-loop and/or surrounding sequences might determine transcriptional termination efficiencies or, in other words, the levels of RNAP read-through that occur at a particular TT [37,38,39]. The analysis of the TT*_sbrB_* sequence revealed that nucleotides A112 and C136 were not paired in the stem-loop of the *S. aureus* 15981 TT*_sbrB_* (Figure 4A). In order to explore whether this mispairing was conserved, we performed BLASTN against representative *S. aureus* genomes. We discovered that the *sbrB* mRNA of our clinical isolate carried a single nucleotide substitution in position 112 (G112A), which produced the TT*_sbrB_* mispairing (Figure 4A). To address whether this single nucleotide change could be responsible for the TT*_sbrB_* read-through, we performed Northern blots and compared the *lexA*-asRNA levels between the *S. aureus* 15981 and MW2 strains. The MW2 strain served as a control as it did not carry the substitution that caused the aforementioned mispairing (Figure 4A). As shown in Figure 4B, *lexA*-asRNA could only be detected in the *S. aureus* 15981 and ΔTT strains (Figure 4B). Since the *sbrB* mRNA was well expressed in both WT strains (Figure 4B), the observed differences could not be attributed to a lower SigB activation in MW2. To validate the implications of the G112A substitution in the transcriptional read-through of the *sbrB* mRNA, we chromosomally corrected the mutation in the *S. aureus* 15981 strain (A112G_15981_). Northern blot results showed that the expression of *lexA*-asRNA was prevented in the A112G_15981_ strain in the same manner as it was occurring in the MW2 strain. These results indicate that the nucleotide mispairing within the TT*_sbrB_* hairpin is responsible for the read-through in the *S. aureus* 15981 strain.

### 2.5. Variations in the TT_sbrB_ Sequence in Other Staphylococcus Strains Produce Different TT_sbrB_ Read-Through Levels

**Figure 5:**
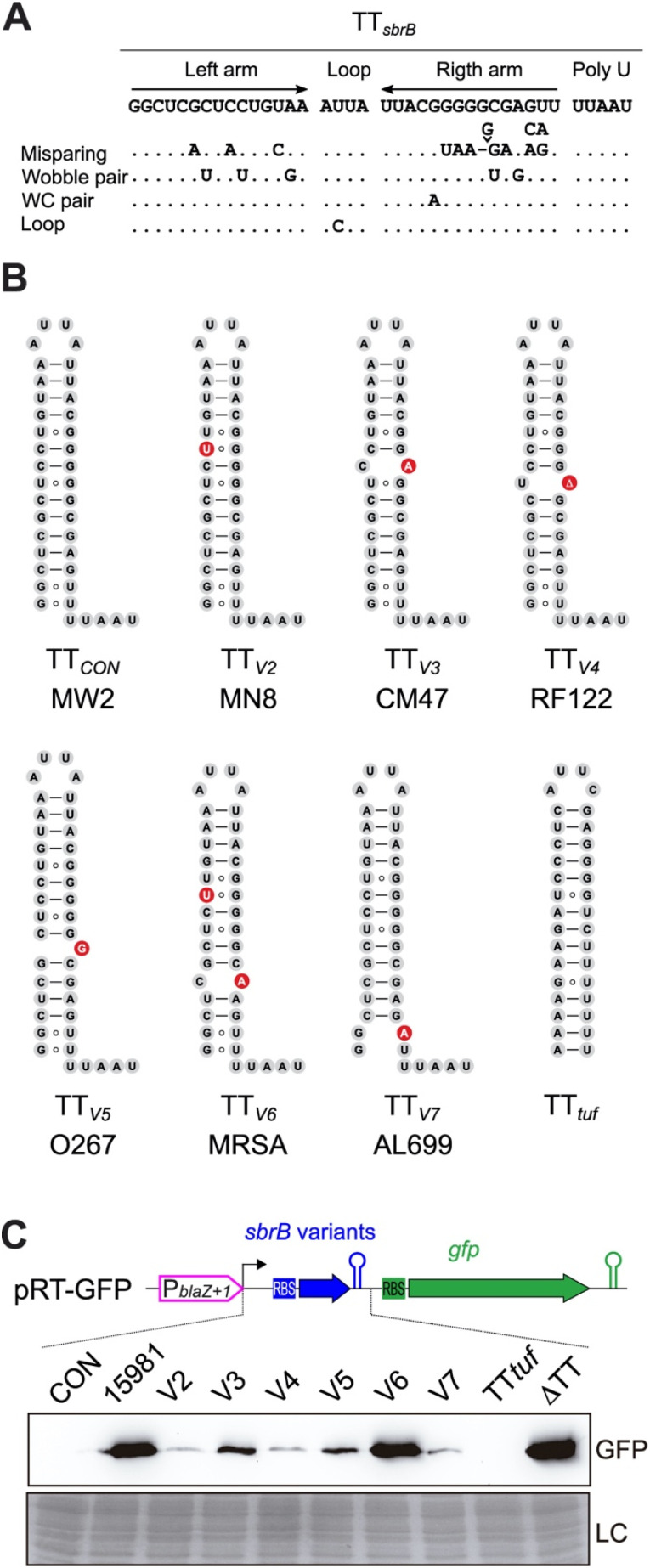
Natural mutations found in the TT*_sbrB_* sequence influence the read-through levels. (**A**) Localization of nucleotide variations found in the TT_sbrB_. The changes in the TT structure produced by the mutations are indicated. (**B**) Putative RNA structures of selected TT*_sbrB_* variants. Mispairing nucleotides are highlighted in red. Frequencies of the mutations among staphylococcal strains are indicated in Table S4. (**C**) Western blot analysis showing the levels of transcriptional read-through (RT) produced by the different nucleotide variations were monitored by measuring the GFP expression. The different *sbrB* mRNA variants were cloned upstream of the *gfp* gene reporter and expressed under the control of the P_*blaZ*+1_ promoter. Total proteins transferred to nitrocellulose membranes were incubated with monoclonal anti-GFP and developed with peroxidase-conjugated goat anti-mouse antibodies and a bioluminescence kit. Coomassie gel portions are included as loading controls (LC).

Safina et al. recently reconstructed the evolutionary history of Rho-independent terminators of the *Bacillus cereus* genes and showed that the nucleotide sequences carrying TT structures are diverse [40]. Although the differences at the sequence level may not reflect a structural change for the majority of cases, in some, mispairing nucleotides may have been evolutionarily selected to offer another mechanism of post-transcriptional regulation [40]. Thus, we wondered whether the *S. aureus* TT*_sbrB_* could also be prone to acquiring natural mutations. BLASTN analyses, using the *sbrB* mRNA as a query, against all the complete and incomplete *S. aureus* genomes available in the NCBI server, revealed that 571 out of 10,399 *S. aureus* genomes presented nucleotide changes in TT*_sbrB_* that involved 21 distinct mutations (Figure 5A and Table S4). Fourteen of these mutations created mispairings in the TT*_sbrB_* stem-loop in a similar fashion to that of our clinical isolate (Table S4). In addition to the G112A mutation (V1) found in *S. aureus* 15981, mutations in the V3 (G132A), V6 (C116U/G136A), V8 (G136A), V14 (G131U) and V19 (C115A) variants considerably affected the thermostability of the TT (Table S4). Additionally, there were seven mutations that changed a Watson–Crick (WC) pair by a Wobble pair in the stem loop, which produced a lower effect on the TT thermostability than the mispairing mutations (Table S4). Interestingly, most of the mutations were concentrated in the right arm of the TT stem-loop (Figure 5A).

In order to evaluate whether these nucleotide variations affected the TT*_sbrB_* read-through levels, we reproduced several of the *sbrB* mRNA natural variants using the pRT transcriptional reporter plasmids (Figure 5B,C). As controls, we included the *S. aureus* 15981 *sbrB* (V1), the *sbrB* mRNA carrying the TT deletion (ΔTT) and a chimeric *sbrB* mRNA carrying the TT from the *tuf* gene instead the original one. TT*_tuf_* was selected because no read-through was observed in our previous analysis [4]. Interestingly, Western blot results showed a palette of GFP expression levels depending on the TT*_sbrB_* sequence. While the TT*_con_* and TT*_tuf_ sbrB* variants did not express any detectable GFP levels, control V1 (*S. aureus* 15981 natural sequence), V6 (which combined two mutations) and the ΔTT plasmid displayed the highest levels of GFP. V3 and V5 showed an intermediate GFP expression profile while the GFP levels were the lowest in the V2, V4 and V7 variants (Figure 5C). Despite this, we were unable to see a direct correlation between the type of mutation and the GFP levels (Figure 5). Nevertheless, these results indicated that natural variations in the TT*_sbrB_* sequence modified the RNAP transcriptional termination efficiency. As a consequence, the variations in the TT*_sbrB_* sequence throughout the different *S. aureus* strains have a critical impact on the generation of *lexA*-asRNA and its expression levels.

Considering that the *sbrB* gene is conserved among other staphylococcal species [28], we extended the BLASTN comparison to the genus *Staphylococcus* (excluding *S. aureus* strains) using the *sbrB* TT*_con_* sequence as a query. No hits could be obtained in other *Staphylococcus* spp genome sequences, indicating that TT*_sbrB_* was not conserved despite the conservation of the *sbrB* gene. A deeper comparative analysis using MAUVE [41] revealed that although the CDS organization in the *sbrB-lexA-sosA* locus was similar, the *sbrB* 3′-end and the *sbrB*-*lexA* intergenic region (IGR) were not conserved among staphylococcal species (Figure S2A). This suggested that this region might have been subjected to changes throughout evolution, a hypothesis that is in agreement with our previous study, which showed an evolutionary bias among 3′UTRs in bacteria [42]. The lack of TT*_sbrB_* conservation in the other *Staphylococcus* species did not mean an absence of TT sequences, since alternative TTs could be predicted using the RNA structure V6.2 software (Figure S2B). To analyze the transcriptional termination efficiencies of these TTs, we cloned the *sbrB* mRNA sequences from *S. epidermidis* RP62A, *S. capitis* SK14 and *S. argenteus* MSHR1132 strains into the pCN57_+1_ plasmid. Western blot results showed that the three *sbrB* species expressed higher GFP levels than the control strains, indicating that these TTs were also affected by read-through events. Interestingly, the TT*_sbrB_* from *S. epidermidis* carried a mispairing nucleotide pair in its stem-loop, which might explain its lower termination efficiency (Figure S2C). Note that, in contrast to *S. aureus*, this mispairing was evolutionarily fixed in other *S. epidermidis* genome sequences available at NCBI database. From these experiments, we concluded that specific TT*_sbrB_* sequences from particular species and strains determine the RNAP transcriptional termination efficiency and, ultimately, the levels of asRNA-*lexA*.

### 2.6. The asRNA/mRNA Ratio Drives the LexA Reporter Expression

**Figure 6:**
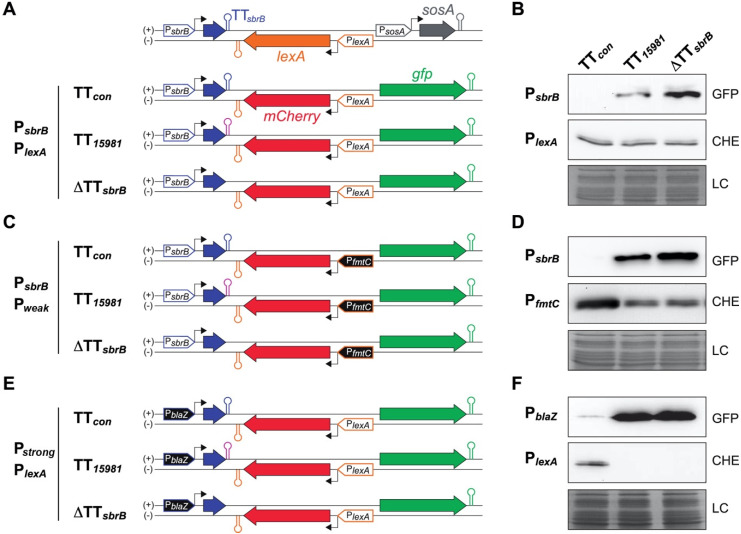
*lexA*-asRNA impacts the mCherry LexA reporter expression depending on the P*_sbrB_* and P*_lexA_* ratio. (**A**,**C**,**E**) Schematic representations of dual fluorescent reporter plasmids carrying the *sbrB-lexA* locus and its corresponding variations. These plasmids monitored the *lexA*-asRNA expression and P*_lexA_* promoter activity by producing GFP and mCherry, respectively. In order to avoid autoregulation, the *lexA* CDS was substituted by the *mCherry* CDS while preserving the 3′- and 5′-UTRs of *lexA* mRNA. When indicated, the P*_sbrB_* and P*_lexA_* promoters were replaced by the P_*blaZ*+1_ and P*_fmtC_* promoters, respectively. For each promoter combination, a dual fluorescent reporter plasmid carrying the TT*_sbrB_* sequences from the *S. aureus* 15981 (TT*_15981_*, pink hairpin) and MW2 (TT*_con_*, blue hairpin) strains and a ΔTT*_sbrB_* mutant was constructed. (**B**,**D**,**F**) Western blot analyses showing the GFP and mCherry levels expressed from the strains carrying the dual reporters shown in (**A**,**C**,**E**). Total proteins were transferred to nitrocellulose membranes and incubated with monoclonal anti-GFP or anti-mCherry antibodies. These were then developed with peroxidase-conjugated goat anti-mouse or goat anti-rabbit antibodies, respectively, and a bioluminescence kit. Coomassie gel portions are included as loading controls (LC).

To investigate whether *lexA*-asRNA affects the expression of *lexA*, we constructed two chromosomal mutant strains by deleting the P*_sbrB_* promoter (ΔP*_sbrB_*) or replacing this promoter by the constitutive P*_blaZ_* promoter (P*_blaZ_-sbrB*) in our clinical isolate, respectively. Northern blot experiments revealed that the *lexA* mRNA seemed unaffected despite the high levels of *lexA*-asRNA expressed in the P*_blaZ_-sbrB* strain (Figure S3A). Similar results were obtained when LexA protein levels were analyzed by Western blotting using specific anti-LexA antibodies (Figure S3B). LexA is known for being heterogeneously expressed within bacterial populations [43,44]. A putative stochastic expression of LexA might mask the effect of the asRNA within the bacterial population. To evaluate this possibility, we designed a dual fluorescent reporter plasmid carrying the *sbrB*-*lexA* region from *S. aureus* 15981, in which the *lexA* CDS was substituted by the *mCherry* CDS while preserving the 3′ and 5′UTRs of *lexA*. (Figure 6A). Therefore, the mCherry expression would be under the control of the *lexA* promoter. Moreover, the absence of the *lexA* CDS would avoid the auto-regulatory effect [45] on the reporter system. The *gfp* reporter gene was placed downstream of the *lexA* promoter, on the forward strand, to monitor the expression of the artificial *mCherry* asRNA. Note that *sosA* promoter was not included in the construction to strictly report the asRNA level (Figure 6A). Based on this construction, we generated two additional variants: one lacking the TT*_sbrB_* sequence (ΔTT*_sbrB_*) and another one carrying the consensus TT*_sbrB_* from the MW2 strain (TT*_con_*) (Figure 6A). In agreement with previous results, Western blot analyses showed that the GFP expression could be detected in the strain carrying the TT*_15981_* and ΔTT*_sbrB_* construct variants. However, no GFP expression was observed in bacterial cells carrying the TT*_con_* variant (Figure 6B). No variations were found in the mCherry expression, indicating that the levels of the asRNA were insufficient to visibly alter the red reporter expression when transcribed from the P*_lexA_* promoter, at least at the bulk population level (Figure 6B). We then generated a new set of plasmid reporters by replacing the P*_lexA_* and P*_sbrB_* promoters with the *fmtC* promoter (P*_weak_*, a weaker promoter showing a ~5-fold lower activity than the former one [4]) (Figure 6C) and the P*_blaZ_* promoter (P*_strong_*, a constitutive stronger promoter than P*_sbrB_*) (Figure 6E), respectively. The expression of mCherry under the control of the *fmtC* promoter was reduced in the TT*_15981_* and ΔTT*_sbrB_* variants when compared to the TT*_con_* variant (Figure 6D), indicating that the *lexA*-asRNA levels could alter the sense mRNA expression when the promoter activity was lower. In the latter, the P*_sbrB_* promoter was substituted by the highly active P*_blaZ_* promoter (Figure 6E). The overexpression of the asRNA resulted in a clear decrease in the mCherry expression, suggesting that the *lexA* mRNA could be inhibited in conditions of high activation of the P*_sbrB_* promoter (Figure 6F). Note that the mCherry expression was unaffected in the reporter plasmids carrying the TT*_con_* (Figure 6D,F). Altogether, these results indicate that the *lexA*-asRNA generated by TT*_sbrB_* mutations could have an impact on the *lexA* mRNA expression depending on the transcriptional ratio of the P*_sbrB_* and P*_lexA_* promoters.

### 2.7. Heterogeneity on the LexA Reporter Expression Is Reduced by lexA-asRNA

**Figure 7:**
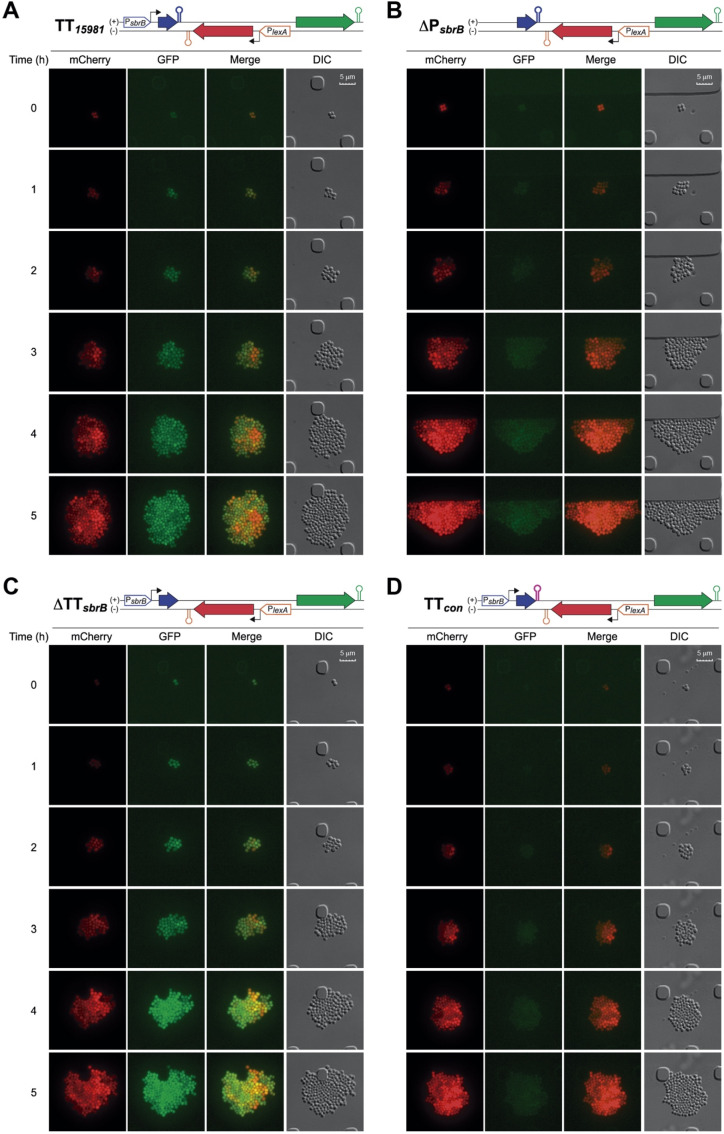
Regulation of heterogenous LexA expression by *lexA*-asRNA. Time-lapse fluorescence microscopy was performed to monitor GFP and mCherry expression at the single-cell level in *S. aureus* 15981 strains carrying one of the four dual fluorescent plasmid reporter variants: (**A**) TT*_15981_*, (**B**) ΔP*_sbrB_*, (**C**) ΔTT*_sbrB_* and (**D**) TT*_con_*. The main genetic elements of these constructions are illustrated on top of each corresponding panel. Bacteria were grown at 37 °C in CellAsic microfluidic plates with a continuous flow of MHg and challenged with 30 mM of KOH for 4 h. Images were taken in 15 min intervals. The mCherry and GFP fluorescence, the combination of both signals (merge panels) and the differential interference contrast (DIC) images are shown. Quantification of bacterial growth and single-cell GFP and mCherry levels are shown in Supplementary Figure S4.

Since it was previously shown that LexA expression varies within bacterial populations [43,44], it was reasonable to expect a similar behavior for *S. aureus* LexA. Therefore, we evaluated the effect of the asRNA on the LexA reporter at the single-cell level by performing time-lapse fluorescence microscopy. We used *S. aureus* strains carrying four dual reporter plasmid variants including the *S. aureus* 15981 TT*_sbrB_* (TT*_15981_*) and the consensus TT*_sbrB_* (TT*_con_*) sequences or the deletion of the *sbrB* terminator (ΔTT*_sbrB_*) and *sbrB* promoter (ΔP*_sbrB_*) sequences as controls (Figure 7). Note that the expressions of the GFP *lexA*-asRNA and mCherry LexA reporters were under the control of the native P*_sbrB_* and P*_lexA_* promoters, respectively (Figure 7 and Figure S4A). These strains were grown in CellAsic microfluidic plates at 37 °C with a continuous flow of MH medium and challenged with KOH for 4 h to induce the P*_sbrB_* promoter. Images were recorded every 15 min and the mCherry and GFP fluorescence intensities of each individual cell from two microcolonies were measured using the ROI statistics plugin of the Icy bioimage software [46]. In these conditions and starting from just a couple of *S. aureus* cells, microcolonies of up to 100–200 cells were obtained (Figure S4B). We found that the mCherry LexA reporter was heterogeneously expressed among individuals (Figure 7). Interestingly, single-cell quantification of mCherry and GFP levels revealed that the variability in the mCherry LexA reporter expression observed in individual cells was higher in the strains that did not express *lexA*-asRNA (TT*_con_* and ΔP*_sbrB_*) (Figure S4C,D). In contrast, in the TT*_15981_* and ΔTT*_sbrB_* strains that expressed the *lexA*-asRNA (Figure S4D), heterogenicity on the mCherry expression was considerably reduced (Figure S4C). These observations suggest that *lexA*-asRNA may act as a gatekeeper, lowering the threshold of LexA expression at the single cell level. This could have a beneficial impact in particular stress situations that activate the SigB-dependent *sbrB* promoter.

### 2.8. Mispairing Nucleotides in Intrinsic Terminators Transcriptionally Connect Contiguous Genes

**Figure 8:**
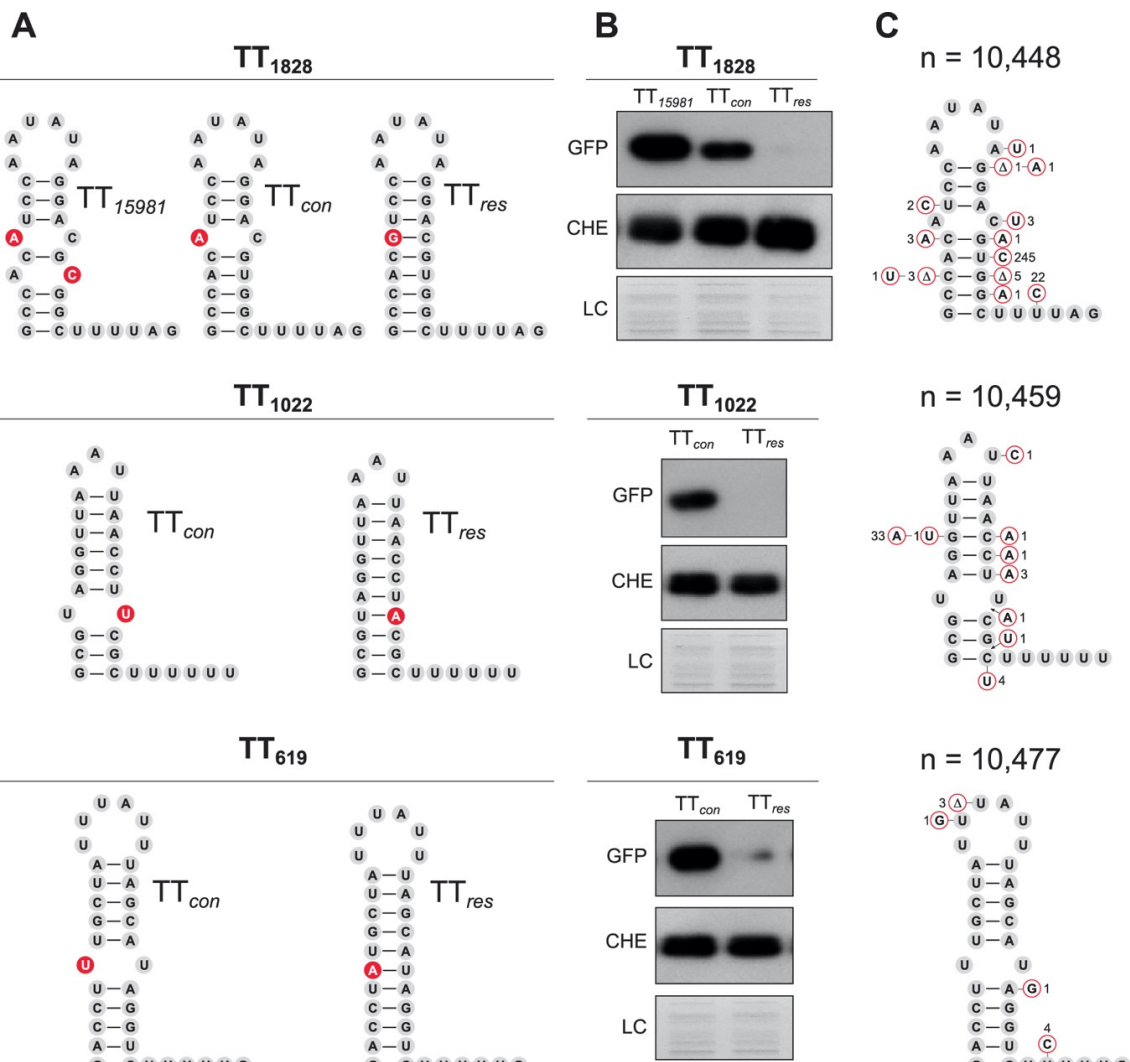
Evolutionarily selected mispairing nucleotides in TTs are relevant for read-through events. (**A**) Structures of predicted TTs (1828, 1022 and 619) including mispairings and restored TT variants (res) that compensated the mispairings. Nucleotide changes are colored in red. (**B**) The TT read-through levels from each variation were monitored by Western blot analyses using the dual fluorescent reporter plasmid shown in Supplementary Figure S6. The GFP expression levels indicate the amount of transcriptional read-through while mCherry monitors the transcript level expressed from the P*_sbrB_* promoter. Proteins transferred to nitrocellulose membranes were developed as indicated in Figure 6. Coomassie-stained gel portions are included as loading controls. (**C**) Identification of natural nucleotide variations in the corresponding TTs. The sequences of the selected TTs were compared by BLASTN against all *S. aureus* genomes available in the NCBI database. n represents the number of genomes compared for each TT. The nucleotide variations and the number of genomes carrying such variations are indicated in the corresponding TT position.

The findings presented here suggest that natural mutations alter the strength of TTs. In order to investigate whether TREs originated by mispairings could also apply to other intrinsic TTs, we performed genome-wide scale analyses. We first predicted the intrinsic TTs in the *S. aureus* NCTC 8325 reference genome using the TransTermHP v2.07 software [47] and classified them according to their genomic position (Figure S5 and Table S5). Then, we determined the level of read-through for each TT using our previous RNA sequencing data from the *S. aureus* 15981 strain [4]. We listed 566 out of 965 predicted TTs (with a confidence interval higher than 75%) having gene expression levels higher than 4 (log_2_) (Table S5). Among them, 85 TTs showed read-through levels between 50% and 100% relative to the gene transcript level (Table S5). Interestingly, 33 out of these 85 TTs presented mispairing nucleotides. To validate whether these mispairings were responsible for TREs, we selected three examples from different categories but with a similar hairpin structure to that of TT*_sbrB_* (TT_1828_, TT_1022_ and TT_619_) (Table S5 and Figure 8). TT_1828_ was located between two convergent genes and the TRE produced 3′UTR overlapping transcripts [8] (Figure S7A), TT_1022_ was located between two CDSs (*fur* and *xerD*) from a polycistronic transcript (Figure S7B) and TT_619_ belonged to the *yybP-ykoY* riboswitch (Figure S7C) [48]. Each TT sequence was introduced into a dual fluorescent reporter plasmid under the control of the P*_sbrB_* promoter (Figure S6A). The mCherry and GFP reporters were used to monitor the transcription upstream and downstream of the TT, respectively. In addition, mutant TTs that compensated for the mispairings of the selected candidates were constructed and cloned in the same vector. The mCherry reporter monitored putative transcript level variations due to modified TT sequences while the GFP addressed the read-through levels (Figure S6A). The functionality of this reporter system was tested using TT*_sbrB_* as control. As expected, TT*_sbrB_* from *S. aureus* 15981 expressed high GFP levels when compared to the consensus TT (TT*_con_*) while both showed similar levels of mCherry (Figure S6B). Similarly, Western blot results revealed that plasmids carrying TT_1828,_ TT_1022_ and TT_619_ expressed high levels of GFPs, indicating that these TTs also suffered TREs. In contrast, when the mispairings were restored (Figure 8A), expression of GFP was abolished, confirming that they were involved on the TRE (Figure 8B).

Further conservation analyses showed that the mispairings were conserved in the selected examples, indicating that they had been evolutionarily selected. Specifically, TT_1022_ and TT_619_ presented significantly fewer variations (46 and 9 out of 10,459 and 10,477, respectively) than TT_1828_, which applied to 289 out of 10,448 genomes (Figure 8C). Among the TT_1828_ variations, the U21C substitution was the most common (245 genomes) (Figure 8C) and it was also present in the *S. aureus* 15981 strain. As shown in Figure 8B, the additional mispairing created by the U21C substitution significantly increased the read-through already present in the TT*_con_*, while the modified TT_1828_ (TT*_res_*), which completely restored the nucleotide pairing, inhibited the TRE (Figure 8B). This TT constituted an example in which both evolutionarily fixed and strain-specific mispairings were combined to modify the TT function. Interestingly, we found three genomes carrying a C19U substitution, which naturally restored the nucleotide pairing (Figure 8C). Altogether, these results support that the termination efficiency of particular TTs could be modified by both evolutionarily selected mispairings and strain-specific natural mutations, resulting in different read-through levels. These mispairings could be a novel source of genetic variability to produce transcriptomic differences among strains.

## 3. Discussion

In this study, we characterized the *lexA* antisense RNA, which we previously identified in the *S. aureus* 15981 clinical isolate [4], and discovered that was transcribed thanks to a natural mutation within the intrinsic terminator of the *sbrB* gene. The G112A substitution generated a mispairing in the TT*_sbrB_* hairpin, which affected the TT efficiency producing a TRE (Figure 4). As a result, *S. aureus* 15981 generated an alternative transcript that originated from the SigB-dependent promoter of the *sbrB* gene and overlapped with the *lexA* mRNA (Figure 1). Our results showed that the mRNA and protein levels of LexA from batch cultures seemed unaffected when *lexA*-asRNA was constitutively expressed (Figure S3). Several hypotheses could explain these results. First, the *lexA* mRNA could be highly expressed in the tested conditions and, therefore, slight variations would be difficult to detect. In fact, when comparing the levels of the *lexA* mRNA and *lexA*-asRNA from previous transcriptomic data, we found them to be 10-fold greater for the former (1307 vs. 149 RPK, respectively) [4]. Second, LexA regulates its own expression [45]; as a consequence, the amount of LexA might be continuously adjusted. Third, LexA could be heterogeneously expressed in *S. aureus* populations, as previously described [43,44]. Our results showed that some of these hypotheses could simultaneously apply. When the *lexA* and *sbrB* promoter activity ratios were altered in absence of the LexA auto-regulation, *lexA*-asRNA could affect the LexA expression reporter at the population level (Figure 6). Moreover, in growth conditions in which SigB and, as a result, the *sbrB* promoter were induced by alkaline stress, *lexA*-asRNA reduced the LexA-reporter levels and its expression variability in single cells (Figure S4). This suggested that the G112A mutation might be a subtle evolutionary advantage that enables the bacterium to modulate LexA expression in particular conditions. This proves interesting since it connects the SOS response and the stress response through a natural single-nucleotide polymorphism. The *lexA*-asRNA transcript also encodes the SbpB and SosA proteins (Figure 1). SosA is proposed to act as a cell division inhibitor that is negatively regulated by LexA [49,50]. Remarkably, in the transcriptional configuration observed for the *S. aureus* 15981 strain, the SosA expression might be activated by *lexA*-asRNA through inhibition of LexA expression and, at the same time, by providing additional mRNAs for SosA translation. This was confirmed by the dual reporter assays that showed GFP expression for constructions carrying the TT*_15981_* sequence even in the presence of an active LexA expression. Note that the GFP CDS is encoded in the opposite DNA strand and downstream of mCherry *lexA* reporter gene. Therefore, this natural mutation could inhibit LexA expression and, hence, represent a new form of SOS response activation. In this scenario, the triggering signal would not be in the form of DNA damage but would come from SigB activating stress signals. It is reasonable to think that this could give *S. aureus* 15981 an adaptive advantage in certain conditions.

In the NCBI database, there are more than 500 *S. aureus* genomes with single-nucleotide polymorphisms within the TT*_sbrB_* sequence (Table S4). We were able to distinguish 21 unique mutations, several of which produced different levels of transcriptional read-through (Figure 5). This suggests that this particular region might be prone to sequence variability that could lead to diversity among *S. aureus* strains. In other species from the genus *Staphylococcus*, such as *S. epidermidis*, *S. capitis* and *S. argenteus*, the *sbrB* TT presented higher read-through levels than the consensus *S. aureus* TT*_sbrB_* (Figure S2). Interestingly, the *S. epidermidis* TT*_sbrB_* contained a mispairing that was evolutionarily fixed, indicating that in this species, the SOS–SigB antisense transcriptional connection might be the norm.

These results led us to question the possibility of environmental pressures, such as antibiotics, favoring the selection of TT*_sbrB_* mutations in *S. aureus*. β-lactam antibiotics are known to activate the SOS response [51]. The *S. aureus* 15981 strain was originally isolated from an otitis infection [52]; therefore, it could be speculated that this strain may probably have been exposed to antibiotic treatments (e.g., β-lactam) prior to its isolation. The SOS response is mechanistically involved in generating mutations that allow the selection of highly oxacillin-resistant populations [53]. Interestingly, this process is modulated by the Agr quorum sensing system. Previous studies showed how the inactivation of *agr* significantly increases the number of rifampin mutants, indicating that the SOS-mediated mutation rate is increased in such conditions [54]. Coincidentally, *agr*-negative variants are commonly isolated from human infections [55]. In fact, *S. aureus* 15981 is *agr*-negative. Therefore, it might be possible for the G112A TT*_sbrB_* mutation to have been selected as a result of the antibiotic pressure and host defense mechanisms. The existence of additional genomes carrying TT*_sbrB_* mutations suggests that this selection effectively occurs in *S. aureus*. However, the correlation of strains origin and sequencing data from the NCBI database has not been trivial and this hypothesis could not be proven. It would be interesting to perform evolutionary studies under the presence of different stressors and evaluate the TT*_sbrB_* mutation rate.

Besides the particular transcriptomic connection between the *sbrB*-*lexA*-*sosA* genes, we also showed that mispairing-dependent TREs are common in *S. aureus* (Table S5). Almost 40% of the predicted TTs that presented read-through levels higher than 50% (relative to the transcript level) contained mispairings. This is a very simple mechanism, through which a single-nucleotide polymorphism of the TT hairpin may generate alternative transcripts that affect the expression of downstream genes. This could be considered as an additional mechanism towards transcriptomic variability [56].

Note that TREs in intrinsic TTs occur more frequently than initially anticipated. Chen et al. showed that out of ~220 TTs from *E. coli*, only a few of them were considered efficient terminators [39]. In other words, the strength of *E. coli* intrinsic terminators is highly variable and most of them suffer transcriptional read-throughs, which is in agreement with our study. This phenomenon is not restricted to intrinsic terminators, since inhibition of Rho activity also promotes read-through in Rho-dependent terminators [15]. Additionally, transcriptional termination could be actively modulated by external factors [57]. For example, RNA-binding proteins can induce transcription elongation by forcing the RNA polymerase to ignore the intrinsic TT signals [58,59,60]. This also applies to Rho-dependent termination, which is susceptible to regulation by sRNAs [61,62,63]. It is noteworthy that in our study (Table S5), there were several predicted TTs that lacked mispairings but also produced high levels of read-through. Whether the stem-loop length, its GC content, the sequence of the poly-U track, the presence of particular RNA-binding proteins and/or a combination of them are involved in the TRE remains to be investigated.

In summary, our study highlights intrinsic TTs as additional key elements for the generation of novel transcriptional architectures while broadening our views on the transcriptomic complexity of bacteria.

## 4. Materials and Methods

### 4.1. Strains, Plasmids, Oligonucleotides and Growth Conditions

The bacterial strains, plasmids and oligonucleotides used in this study are listed in Supplementary Tables S1–S3, respectively. *Staphylococcus aureus* strains were grown in Mueller Hinton Broth supplemented with 1.25% glucose (MHg), whereas *Escherichia coli* was grown in LB broth. The B2 (casein hydrolysate, 10 g/L; yeast extract, 25 g/L; NaCl, 25 g/L; K_2_HPO_4_, 1 g/L; glucose, 5 g/L; pH 7.5) and SuperBroth (tryptone, 30 g/L; yeast extract, 20 g/L; MOPS, 10 g/L; pH 7) media were used to prepare *S. aureus* and *E. coli* competent cells, respectively. For selective growth, media were supplemented with the appropriate antibiotics at the following concentrations: Erythromycin (Erm), 1.5 μg/mL or 10 μg/mL; Ampicillin (Amp), 100 μg/mL. To induce alkaline stress, 30 mM of KOH was added to the medium.

### 4.2. Generation of Chromosomal Mutants by Homologous Recombination

The mutant strains were obtained by marker-less homologous recombination, using the pMAD plasmid [64] as previously described [65]. Briefly, mutant strains were generated by a two-step recombination process that exchanges a gene of interest by the corresponding mutant allele contained within the pMAD plasmids (Table S2) [52]. The resulting mutant strains were verified by PCR using the oligonucleotides listed in Table S3 and the resulting amplicons Sanger-sequenced for final confirmation.

### 4.3. RNA Extraction and Northern Blotting

Preinocula were grown in 5 mL MHg and incubated overnight at 37 °C and 200 rpm. Bacterial precultures were normalized and diluted 1/100 in Erlenmeyer flasks containing MHg and 10 μg/mL erythromycin when required. Normalized bacterial cultures were incubated at 37 °C and 200 rpm until the exponential phase (OD_600_ 0.5) was reached. Cells were harvested by centrifugation for 3 min at 4400× *g* and 4 °C. Pellets were then frozen in liquid nitrogen and stored at −80 °C until needed. RNA extractions and Northern blots were performed as previously described [66]. Next, 10 μg of total RNA extracts were transferred to 0.2 µm pore size Nitran N membranes (Cytiva, Marlborough, MA, USA) and developed using ^32^P radioactive-labeled riboprobes. These probes were synthesized from PCR products harboring the T7 promoter following the recommendations of the Invitrogen MAXIscript T7 transcription kit (Thermo Fisher Scientific, Waltham, MA, USA). The PCR products were obtained using the following oligonucleotides: LB77 and LB78 for the asRNA probe; sRNA antilexA fw2 and sRNA antilexA T7 for the sbrB probe; LB79 and LB80 for the lexA probe; and sosA fw1 and sosA T7 for the sosA probe (Table S3). RNA probes were radio-labelled with ^32^P-α-UTP following the manufacturer’s recommendations and purified with Amersham MicroSpin G-50 columns (Cytiva, Marlborough, MA, USA). Membranes were hybridized with the corresponding riboprobes at 68 °C overnight and washed three times with preheated 2X SCC, 0.1% SDS followed by at least two washing steps with 0.1X SCC, 0.1% SDS at room temperature until the background signal was non-detectable. Membranes were developed by autoradiography for different time periods.

### 4.4. Simultaneous Mapping of the 5′ and 3′ Ends (mRACE)

Mapping of the transcript 5′ and 3′ ends were performed using the Rapid Amplification of cDNA Ends (mRACE) protocol previously described [31], with the following modifications [42]. Total RNA samples were incubated with the Cap-Clip Acid Pyrophosphatase (Tebu-Bio, Le-Perray-en-Yvelines, France) for 1 h following the manufacturer’s recommendations. RNAs were then treated with phenol-chloroform and precipitated with sodium acetate and cold ethanol. Dilutions of treated and non-treated RNAs were ligated overnight at 16 °C using the T4 RNA Ligase I (New England Biolabs, Ipswich, MA, USA). The SuperScript^TM^ III One-Step RT-PCR System and the Platinum Taq DNA polymerase (Invitrogen, Thermo Fisher Scientific, Waltham, MA, USA) were used for RT-PCR. Oligonucleotides LB75-OP-A and LB76-OP-B (Table S3) were required for amplification, and the resulting PCR products were run in 2.5% agarose gels. The bands of expected size were excised, purified and ligated into the pJET 1.2 cloning vector (Thermo Fisher Scientific, Waltham, MA, USA). Plasmids extracted from five isolated colonies and plasmid inserts were Sanger sequenced. Transcript boundaries were determined by sequence alignments to the corresponding genome reference.

### 4.5. Plasmid Construction

The plasmids used in this study (Table S2) were engineered as previously described [67]. All constructs were verified by Sanger sequencing and electroporated into *S. aureus* competent cells as previously described [68].

The pMAD plasmids (pMAD-ΔP*_sbrB_*, pMAD-ΔTT, pMAD-P_*blaZ*+1_ and pMAD-A112G) carrying the mutant alleles required for the chromosomal modifications were constructed as follows. Briefly, the flanking regions from the target genes of the *S. aureus* 15981 strain were amplified using the specific oligonucleotides listed in Supplementary Table S3. The resulting PCR fragments were cloned into the pJET plasmid. Once the resulting plasmids were purified, the corresponding DNA fragments were excised with restriction enzymes and inserted into the pMAD vector.

The pCN57_+1_ plasmid was constructed to constitutively express the *sbrB* transcript from its native transcriptional start site (TSS). We first mapped the original TSS of the P*_blaZ_* promoter from the pCN57-*sbrB* plasmid. This plasmid was constructed by amplifying the *sbrB* gene from the *S. aureus* 15981 chromosome using oligonucleotides LB35 and LB12 (Table S3). The resulting PCR fragment was ligated into the pCN57 plasmid [69] using PstI and SmaI restriction enzymes. Then, the *S. aureus* 15981 strain was transformed by pCN57-*sbrB* plasmid (Table S2). The 5′ and 3′ ends of the *sbrB* mRNA expressed from the P*_blaZ_* promoter were mapped by mRACE as described above. Once the TSS was identified, the P*_blaZ_* promoter region plasmid and the multiple cloning site (MCS) from the pCN57 plasmid were modified to include an EcoRI restriction site upstream of the P*_blaZ_* TSS. For this purpose, overlapping PCRs were performed using the LB106-LB51 and LB73-LB74 oligonucleotides, respectively, (Table S3) and the pCN57 plasmid as a template. The resulting fragment was excised with SphI and NcoI and ligated into pCN57, generating the modified pCN57_+1_ plasmid (Table S2). The pCN57_+1_-*sbrB* plasmid variant was constructed by PCR amplification using the LB88 and LB12 primers (Table S3) and the *S. aureus* 15981 chromosome as a template. The PCR fragment was ligated into an EcoRI-SmaI digested pCN57_+1_ plasmid (Table S2).

The pHRR plasmid harbors the *mCherry* gene optimized for its expression in *S. aureus* under the control of the Phyper promoter. The codon usage was optimized for *S. aureus* and the *Sa*-*mCherry* gene was produced by gene synthesis at GeneArt (Invitrogen, Thermo Fisher Scientific, Waltham, MA, USA). The *gfp* gene of the pHRG plasmid [70] (Table S2) was replaced by the optimized *mCherry* gene using the SpeI and AscI restriction enzymes, generating the pHRR plasmid.

The pCN56-P*_sbrB_*-CHE plasmid was generated by amplifying the *mCherry* gene from the pHRR plasmid with the LB158 and LB93 oligonucleotide pairs (Table S3). The amplified PCR product was excised using the SphI and BamHI enzymes and ligated into a previously excised pCN56 plasmid (Table S2).

The pP*_sbrB_* transcriptional reporter was constructed using the SalI_sigB_prom and BamHI_GFP_end oligonucleotides (Table S3) and the pAD-cGFP plasmid (Table S2) [71] as a template. The DNA fragment was excised from pJET with SalI and BamHI and ligated into the pCN47 plasmid (Table S2). For the construction of the pP*_sbrB_*-RT transcriptional read-through reporter, oligonucleotides LB1 and LB12 were used to amplify the *sbrB* region that included the *sbrB* promoter and the *sbrB* mRNA from the *S. aureus* 15981 strain. The PCR product was digested with SphI and SmaI and ligated into a previously digested pCN57 plasmid.

The pTL WT translational reporter plasmid was engineered by performing a PCR amplification using the LB51 and LB2 oligonucleotide pair (Table S3) and the pCN57_+1_-*sbrB* plasmid (Table S2) as a template. The resulting amplicon was digested with the SphI and SpeI restriction enzymes and inserted into the pHRG plasmid (Table S2). The pTL RBS, pTL AUG_1_, pTL AUG_2_, pTL AUG_1+2_, pTL STOP_34_ and pTL STOP_58_ plasmids were constructed by amplifying the *sbrB* CDS from pCN57+1-*sbrB* using corresponding oligonucleotide pairs (Table S3). The DNA fragments were digested with EcoRI/SphI and SpeI and ligated into pTL WT (Table S2). Following the same strategy, the pRT WT was created by amplifying the pCN57_+1_-*sbrB* template with LB101 and LB12 (Table S3), digested with EcoRI and SmaI and ligated into the pCN57_+1_ plasmid. The variants of pRT plasmid were obtained by using the corresponding pairs of oligonucleotides (Table S3) and ligating the resulting amplicons into the pRT WT plasmid.

The pTT_15981_-P*_sbrB_*-P*_lexA_* plasmid was constructed by PCR amplification of the *mCherry* gene from the pHRR plasmid (Table S2) using oligonucleotides LB114 and LB93 (Table S3), and the *sbrB* gene from the 15981 chromosomes with primers LB1 and LB92 (Table S3). The PCR products were digested with BamHI-KpnI and SphI-BamHI, respectively, and cloned into the pCN56 plasmid. The pTT_15981_-P*_sbrB_*-P*_fmtC_* plasmid was generated by amplifying the *fmtC* promoter from the *S. aureus* 15981 chromosome using oligonucleotides LB155 and LB154 and cloning the digested (SpeI and KpnI) PCR product into the pTT_15981_-P*_sbrB_*-P*_lexA_* plasmid. The pTT_15981_-P*_blaZ_*-P*_lexA_* was created by excising *P_blaZ_* from the pCN57+1-*sbrB* plasmid using SphI and MluI and ligating the resulting DNA fragment into the pTT_15981_-P*_sbrB_*-P*_lexA_* plasmid. The pTT_con_ and pΔTT variants of these plasmids were originated by performing a PCR of pTT_15981_ using the LB1-LB129 and LB128-LB92 and LB1-LB17 and LB18-LB92 oligonucleotide pairs, respectively (Table S3). Then, oligonucleotides LB1 and LB92 (Table S3) were used for overlapping PCRs. The PCR products were digested with the SphI and BamHI restriction enzymes and ligated into the corresponding pTT plasmid (Table S2).

The pΔP*_sbrB_*-TT_15981_-P*_lexA_* plasmid was engineered by amplifying a DNA fragment from the pTT_15981_-P*_sbrB_*-P*_lexA_* plasmid (Table S2) using primers LB64 and LB92 (Table S3) followed by restriction enzyme digestion with SphI and BamHI. The excised product was ultimately ligated into the pTT_15981_pP*_blaZ_*-P*_lexA_* plasmid (Table S2).

The p*P_sbrB_-che*-TT_15981_-*gfp* plasmid was created by amplifying the pCN57+1-*sbrB* vector with oligonucleotides LB159 and LB160. Then, digestion of the PCR fragment and pCN56-P*_sbrB_*-CHE with BamHI and AscI was performed and both excised fragments were ligated. The p*P_sbrB_-che*-TT*_CON_*-*gfp*, p*P_sbrB_-che*-TT_619_-*gfp* and p*P_sbrB_-che*-TT_1828_-*gfp* plasmids including the consensus TTs and their respective mispairing-restored TTs were generated by PCR amplification using the corresponding oligonucleotide pairs shown in Table S3 and the *S. aureus* 15981 chromosome as a template. The PCRs products were digested with the corresponding restriction enzymes and ligated into the p*P_sbrB_-che*-TT_15981_-*gfp* plasmid.

### 4.6. Bacterial Cultures for Total Protein Extraction and Western Blotting

Bacterial preinocula were grown at 37 °C and 200 rpm overnight. Cultures were normalized, diluted 1:250 in Erlenmeyer flasks containing MHg and 10 μg/mL erythromycin and incubated at 37 °C and 200 rpm until the exponential phase (OD_600_ 0.5) was reached. Bacteria were harvested by centrifugation for 10 min at 4400× *g* and 4 °C. Bacterial pellets were stored at –80 °C until required. Total protein extractions and Western blots were performed as previously described [65]. Briefly, total proteins were run in duplicate 12% polyacrylamide gels. One gel was transferred to a nitrocellulose membrane and incubated with the corresponding antibody. The second gel was stained with Coomassie for loading controls (LC). GFP was developed with a 1:5000 dilution of monoclonal anti-GFP antibodies (Living Colors, Clontech, Takara Bio Inc., Kusatsu, Japan) and peroxidase-conjugated goat anti-mouse immunoglobulin G and M antibodies (1:2500) (Pierce, Thermo Fisher Scientific, Waltham, MA, USA). mCherry was developed using anti-mCherry antibodies (1:1000) and peroxidase-conjugated goat anti-rabbit (1:5000). LexA was developed using polyclonal anti-LexA antibodies (1:5000) and peroxidase-conjugated goat anti-rabbit (1:5000). Membranes were developed in a ChemiDoc Imaging system using the SuperSignal West Pico Chemiluminiscent Subtrate kit (Thermo Fisher Scientific, Waltham, MA, USA).

### 4.7. Time-Lapse Fluorescence Microscopy

Quantification of the GFP and mCherry reporter expression was carried out by time-lapse fluorescence microscopy using microfluidic plates that allow bacterial growth in 2D. The reporter strains were grown until the exponential phase (OD = 0.4) in MHg media and loaded into B04A microfluidic plates (ONIX, CellASIC, Merck, Darmstadt, Germany) following the manufacturer’s instructions. Microfluidic plates were placed into the OKO-lab chamber of a fully automatized Leica DMi8 fluorescence microscope and incubated at 37 °C. The MHg media flow rate was maintained at 2 psi for 1 h and switched to MHg supplemented with 30 mM KOH at 2 psi until the end of the image acquisition. Media switching and flow rate settings were controlled using the CellASIC ONIX FG Software (v 5.5.1.0). Images from four different fields of each microfluidic chamber were acquired every 15 min using a Leica HCX PL APO 100x/1.40–0.70 Oil objective and the LAS X software for 5 h. Images from the GFP, mCherry and differential interference contrast (DIC) channels were processed using the Icy bioimage software [46] (http://icy.bioimageanalysis.org, accessed on 8 December 2021). The GFP and mCherry levels for every single cell were quantified using the ROI statistics plugin of the Icy software.

### 4.8. Determination of Transcriptional Read-Through on Predicted TTs

In silico predictions of intrinsic Rho-independent transcriptional terminators were performed using the TransTermHP v2.07 program as previously described [47]. The *S. aureus* NCTC 8325 genome (NC_007795.1) was used as a reference. The list of predicted TTs was curated by deleting duplicated TTs and predictions with a confidence lower than 75%. The ΔG of the remaining TTs was calculated using the Quickfold program [72]. TTs were also classified according to their genomic positions. To determine the level of read-through for each predicted TT, we counted the RNA-Seq reads upstream and downstream of the TTs using our previous RNA sequencing data from the *S. aureus* 15981 strain [4]. The mean reads/nt of the first 50 nucleotides downstream of a TT was normalized against the mean reads/nt mapping of the first 50 nucleotides upstream of a TT. The latter value represents the transcription level of a gene, while the former indicates the read-through level. For further analyses, we selected 566 out of 965 predicted TTs, which showed hairpin lengths higher than 8 bp and gene transcript levels higher than 4 (log_2_). Subsequently, to identify putative mispairing nucleotides in the predicted TTs, we visually inspected the TT structures generated by the RNAfold algorithm in the Geneious Prime software (Biomatters Ltd., Auckland, New Zealand). The mispairings could be generated either by nucleotide substitutions or single nucleotide insertions/deletions (indels) that disrupted the nucleotide interactions of the hairpin. All of the generated data are compiled in Supplementary Table S5.

## Data Availability

Any data or material that support the findings of this study can be made available by the corresponding author upon request.

## Supplementary Materials

The following supporting information can be downloaded at: https://www.mdpi.com/article/10.3390/ijms23010576/s1.
