# Supplementary material for: Regulation of Heterogenous LexA Expression in Staphylococcus aureus by an Antisense RNA Originating from Transcriptional Read-Through upon Natural Mispairings in the sbrB Intrinsic Terminator
Source: Int J Mol Sci. 2022 Jan 5;23(1):576. doi: 10.3390/ijms23010576 (PMC8745188; doi:10.3390/ijms23010576)
Supplement: Supplementary file 1 [file ijms-23-00576-s001.zip › Sup_Data_Bastet_et_al_v6.pdf]

Supplementary Data for

Regulation of heterogenous LexA expression in *Staphylococcus aureus* by an antisense RNA originating from transcriptional read-through upon natural mispairings in the *sbrB* intrinsic terminator

Laurène Bastet[1], Pilar Bustos-Sanmamed[1], Arancha Catalan-Moreno[1], Carlos J. Caballero[1], Sergio Cuesta[1], Leticia Matilla-Cuenca[1], Maite Villanueva[1], Jaione Valle[1], Iñigo Lasa[2] and Alejandro Toledo-Arana[1]*

[1] Instituto de Agrobiotecnología (IdAB). Consejo Superior de Investigaciones Científicas (CSIC)-Gobierno de Navarra. 31192-Mutilva, Navarra, Spain.

[2] Laboratory of Microbial Pathogenesis. Navarrabiomed-Universidad Pública de Navarra (UPNA)-Complejo Hospitalario de Navarra-IdiSNA. 31008-Pamplona, Navarra, Spain

* To whom correspondence should be addressed:
Alejandro Toledo-Arana
E-mail: a.toledo.arana@csic.es
Phone: +34 948 16 9752
Instituto de Agrobiotecnología, CSIC-GN
Avda. de Pamplona 123, 31192-Mutilva, Navarra
Spain

This PDF file includes:

Supplementary Tables S1 to S5

Supplementary Figures S1 to S7

Supplementary References

Note: Table S5 is provided as a Supplementary Excel file.

Table S1. Strains used in this study

Strains	Relevant characteristic(s)	BGR ID[a]	Reference
Staphylococcus aureus			
15981	Wild type (WT) strain. MSSA clinical isolate; biofilm positive.	8	(1)
MW2	Community-acquired MRSA strain, isolated in 1998 in North Dakota, USA.	10	(2)
ΔTT*sbrB*	WT strain carrying a deletion of the *sbrB* transcriptional terminator (TT)	1104	This study
Δ*sigB*	WT strain carrying a deletion of the *sigB* gene	178	(1)
WT pP*sbrB*	WT strain carrying the pP*sbrB* plasmid	168	This study
Δ*sigB* pP*sbrB*	Δ*sigB* strain carrying the pP*sbrB* plasmid	170	This study
WT pP*sbrB* RT	WT strain carrying the pP*sbrB* RT plasmid	573	This study
Δ*sigB* pP*sbrB* RT	Δ*sigB* strain carrying the pP*sbrB* RT plasmid	665	This study
WT pTL WT	WT strain carrying the pTL WT plasmid	938	This study
WT pTL mRBS	WT strain carrying the pTL mRBS plasmid	948	This study
WT pTL $mAUG_1$	WT strain carrying the pTL $mAUG_1$ plasmid	1001	This study
WT pTL $mAUG_2$	WT strain carrying the pTL $mAUG_2$ plasmid	939	This study
WT pTL $mAUG_{1+2}$	WT strain carrying the pTL $mAUG_{1+2}$ plasmid	1102	This study
WT pTL $STOP_{34}$	WT strain carrying the pTL $STOP_{34}$ plasmid	947	This study
WT pTL $STOP_{58}$	WT strain carrying the pTL $STOP_{58}$ plasmid	1014	This study
WT pRT WT	WT strain carrying the pRT WT plasmid	322	This study
WT pRT mRBS	WT strain carrying the pRT mRBS plasmid	905	This study
WT pRT $mAUG_1$	WT strain carrying the pRT $mAUG_1$ plasmid	951	This study
WT pRT $mAUG_{1+2}$	WT strain carrying the pRT $mAUG_{1+2}$ plasmid	1063	This study
WT pRT $STOP_{34}$	WT strain carrying the pRT $STOP_{34}$ plasmid	904	This study
WT pRT $STOP_{58}$	WT strain carrying the pRT $STOP_{58}$ plasmid	936	This study
WT pRT 31+TT	WT strain carrying the pRT 31+TT plasmid	937	This study
WT pRT ΔTT	WT strain carrying the pRT ΔTT plasmid	895	This study
A112G	WT strain carrying the A112G substitution in the *sbrB* TT	1151	This study
WT pRT-TT_{CON}	WT strain carrying the pRT-TT_{CON} plasmid	943	This study
WT pRT-V2	WT strain carrying the pRT-V2 plasmid	1035	This study
WT pRT-V3	WT strain carrying the pRT-V3 plasmid	1387	This study
WT pRT-V4	WT strain carrying the pRT-V4 plasmid	1388	This study
WT pRT-V5	WT strain carrying the pRT-V5 plasmid	1034	This study
WT pRT-V6	WT strain carrying the pRT-V6 plasmid	1389	This study
WT pRT-V7	WT strain carrying the pRT-V7 plasmid	1390	This study
WT pRT-TT*tuf*	WT strain carrying the pRT-TT*tuf* plasmid	1177	This study
WT pRT-S.arg	WT strain carrying the pRT-S.arg plasmid	1084	This study
WT pRT-S.cap	WT carrying the pRT-S.cap plasmid	1068	This study
WT pRT-S.epi	WT strain carrying the pRT-S.epi plasmid	1067	This study
$ΔP_{sbrB}$	WT strain carrying a deletion of the *sbrB* promoter	180	This study
P_{blaZ}	WT strain carrying the substitution of the *sbrB* promoter by the P*blaZ*$_{+1}$ promoter	1483	This study
WT pTT_{con}- P*sbrB*-P*lexA*	WT strain carrying the pTT_{con}- P*sbrB*-P*lexA* plasmid	999	This study
WT pTT_{15981}-P*sbrB*-P*lexA*	WT strain carrying the pTT_{15981}-P*sbrB*-P*lexA* plasmid	955	This study
WT $pΔTT_{sbrB}$-P*sbrB*-P*lexA*	WT strain carrying the $pΔTT_{sbrB}$-P*sbrB*-P*lexA* plasmid	953	This study
WT pTT_{con}-P*sbrB*-P*fmtC*	WT strain carrying the pTT_{con}-P*sbrB*-P*fmtC* plasmid	1178	This study
WT pTT_{15981}-P*sbrB*-P*fmtC*	WT strain carrying the pTT_{15981}-P*sbrB*-P*fmtC* plasmid	1179	This study

Continued in the following page

Table S1. *Continued*

Strains	Relevant characteristic(s)	BGR ID[a]	Reference
WT pΔTT$_{sbrB}$-P*sbrB*-P*fmtC*	WT strain carrying the pΔTT$_{sbrB}$-P*sbrB*-P*fmtC* plasmid	1181	This study
WT pTT$_{con}$-P*blaZ*-P*lexA*	WT strain carrying the pTT$_{con}$-P*blaZ*-P*lexA* plasmid	1382	This study
WT pTT$_{15981}$pP*blaZ*-P*lexA*	WT strain carrying the pTT$_{15981}$pP*blaZ*-P*lexA* plasmid	1361	This study
WT pΔTT$_{sbrB}$ -pP*blaZ* P*lexA*	WT strain carrying the pΔTT$_{sbrB}$ - pP*blaZ* P*lexA* plasmid	1111	This study
WT pΔP*sbrB*- TT$_{15981}$P*lexA*	WT strain carrying the pΔP*sbrB*- TT$_{15981}$P*lexA* plasmid	957	This study
WT pP*sbrB*-*che*-TT$_{con}$-*gfp*	WT strain carrying the pP*sbrB*-*che*-TT$_{con}$-*gfp* plasmid	1424	This study
WT pP*sbrB*-*che*-TT$_{15981}$-*gfp*	WT strain carrying the pP*sbrB*-*che*-TT$_{15981}$-*gfp* plasmid	1423	This study
WT pP*sbrB*-*che*-TT1828 $_{con}$-*gfp*	WT strain carrying the pP*sbrB*-*che*-TT1828$_{con}$-*gfp* plasmid	1528	This study
WT pP*sbrB*-*che*-TT1828$_{15981}$-*gfp*	WT strain carrying the pP*sbrB*-*che*-TT1828$_{15981}$-*gfp* plasmid	1527	This study
WT pP*sbrB*-*che*-TT1828 $_{res}$-*gfp*	WT strain carrying the pP*sbrB*-*che*-TT1828$_{res}$-*gfp* plasmid	1705	This study
WT pP*sbrB*-*che*-TT1022$_{con}$-*gfp*	WT strain carrying the pP*sbrB*-*che*-TT1022$_{con}$-*gfp* plasmid	1557	This study
WT pP*sbrB*-*che*-TT1022$_{res}$-*gfp*	WT strain carrying the pP*sbrB*-*che*-TT1022$_{res}$-*gfp* plasmid	1558	This study
WT pP*sbrB*-*che*-TT619$_{con}$-*gfp*	WT strain carrying the pP*sbrB*-*che*-TT619$_{con}$-*gfp* plasmid	1793	This study
WT pP*sbrB*-*che*-TT619$_{res}$-*gfp*	WT strain carrying the pP*sbrB*-*che*-TT619$_{res}$-*gfp* plasmid	1801	This study
Escherichia coli			
XL1-Blue	Strain used for cloning experiments	1	Stratagene

[a] Identification number of the strains stored at the Laboratory of Bacterial Gene Regulation

Table S2. Plasmids used in this study

Plasmids	Relevant characteristic(s)	Reference
pMAD	*E. coli-S. aureus* shuttle vector with a thermosensitive origin of replication for Gram-positive bacteria. Amp^R, Erm^R	(3)
pMAD-A112G	pMAD containing the allele for introducing the A122G substitution in the *sbrB* TT	This study
pMAD-ΔTT	pMAD containing the allele for deletion of the *sbrB* TT	This study
pCN57	*E. coli-S. aureus* shuttle vector to express genes under the control of the P*blaZ* promoter and for transcriptional fusions with *gfp* reporter gene. Low copy number. Amp^R-Erm^R	(4)
pCN57-*sbrB*	pCN57 plasmid expressing the *sbrB* mRNA	This study
pCN57+1	pCN57 plasmid carrying the modified *PblaZ* promoter region to express mRNAs from their native transcriptional start site.	This study
pCN57+1-*sbrB*	pCN57+1 plasmid expressing the *sbrB* mRNA	This study
pCN47	*E. coli-S. aureus* shuttle vector. Low copy number. Amp^R-Erm^R	(4)
pHRG	pCN47 plasmid carrying the P*hyper* constitutive promoter, *icaR* RBS and the *gfp* reporter gene	(5)
pHRR	pCN47 plasmid carrying the P*hyper* constitutive promoter, *icaR* RBS and the *mCherry* reporter gene	This study
pCN56	*E. coli-S. aureus* shuttle vector used for transcriptional fusions with the *gfp* reporter. High copy number. Amp^R-Erm^R	(4)
pAD-cGFP	pPL2-P*hyper*-GFP	(6)
pP*sbrB*	pCN47 plasmid carrying the *sbrB* promoter fused to the *gfp* reporter gene	This study
pP*sbrB* RT	pCN57 plasmid expressing the *sbrB* mRNA under the control of its own promoter and cloned upstream the *gfp* reporter gene	This study
pTL WT	pHRG plasmid in which P*hyper* was replaced by *Pblaz+1* and the *sbrB* coding sequence was fused to the ATG-less *gfp* reporter gene	This study
pTL mRBS	pTL WT plasmid where the guanines at position 5 and 6 were substituted by cytosines.	This study
pTL $mAUG_1$	pTL WT plasmid where the thymine at position 20 was substituted by an adenine.	This study
pTL $mAUG_2$	pTL WT plasmid where the thymine at position 23 was substituted by an adenine.	This study
pTL $mAUG_{1+2}$	pTL WT plasmid where the thymines at position 20 and 23 were substituted by adenines.	This study
pTL $STOP_{34}$	pTL WT plasmid where the adenine at position 34 was substituted by a thymine.	This study
pTL $STOP_{58}$	pTL WT plasmid where the guanine at position 58 was substituted by a thymine.	This study
pRT WT	pCN57+1 plasmid expressing the *sbrB* mRNA	This study
pRT mRBS	pRT WT plasmid where the guanines at position 5 and 6 were substituted by cytosines.	This study
pRT $mAUG_1$	pRT WT plasmid where the thymine at position 20 was substituted by an adenine.	This study
pRT $mAUG_{1+2}$	pRT WT plasmid where the thymines at position 20 and 23 were substituted by adenines.	This study
pRT $STOP_{34}$	pRT WT plasmid where the adenine at position 34 was substituted by a thymine.	This study
pRT $STOP_{58}$	pRT WT plasmid where the guanine at position 58 was substituted by a thymine.	This study

Continued in the following page

Table S2. *Continued*

Plasmids	Relevant characteristic(s)	Reference
pRT 31+TT	pRT WT with an insertion of 31 nt between the STOP codon and the TT	This study
pRT ΔTT	pRT WT plasmid where the *sbrB* TT has been deleted	This study
pRT-TT_{CON}	$pCN57_{+1}$ plasmid carrying the *sbrB* mRNA and the MW2 *sbrB* TT	This study
pRT-V2	$pCN57_{+1}$ plasmid carrying the *sbrB* mRNA and the V2 *sbrB* TT	This study
pRT-V3	$pCN57_{+1}$ plasmid carrying the *sbrB* mRNA and the V3 *sbrB* TT	This study
pRT-V4	$pCN57_{+1}$ plasmid carrying the *sbrB* mRNA and the V4 *sbrB* TT	This study
pRT-V5	$pCN57_{+1}$ plasmid carrying the *sbrB* mRNA and the V5 *sbrB* TT	This study
pRT-V6	$pCN57_{+1}$ plasmid carrying the *sbrB* mRNA and the V6 *sbrB* TT	This study
pRT-V7	$pCN57_{+1}$ plasmid carrying the *sbrB* mRNA and the V7 *sbrB* TT	This study
pRT-TT*tuf*	$pCN57_{+1}$ plasmid carrying the *sbrB* mRNA in which the *sbrB* TT was substituted by the *tuf* TT	This study
pRT-S.arg	$pCN57_{+1}$ plasmid carrying the *sbrB* mRNA, whose sequence was cloned from the *S. argenteus* MSHR1132 genome	This study
pRT-S.cap	$pCN57_{+1}$ plasmid carrying the *sbrB* mRNA, whose sequence was cloned from the *S. capitis* SK14 genome	This study
pRT-S.epi	$pCN57_{+1}$ plasmid carrying the *sbrB* mRNA, whose sequence was cloned from the *S. epidermidis* RP62A genome	This study
pMAD_ΔPsbrB	pMAD containing the allele for deletion of the *sbrB* promoter	This study
pMAD_$PblaZ_{+1}$	pMAD containing the allele for substitution of the *sbrB* promoter by P_{blaZ+1}	This study
pTT_{con}- P*sbrB*-P*lexA*	pCN56 plasmid carrying a dual fluorescent reporter that mimics the *sbrB-lexA-sosA* region to simultaneously monitor the expression of LexA and *lexA*-asRNA. The *lexA* and *sosA* genes were replaced by the *mcherry* and *gfp*, respectively. The P*sosA* promoter was excluded to only monitor the *lexA*-asRNA expression. This construction includes the consensus *sbrB* TT	This study
pTT_{15981}-P*sbrB*-P*lexA*	pTT_{con}- P*sbrB*-P*lexA* carrying the 15981 *sbrB* TT instead of the consensus *sbrB* TT	This study
pΔTT_{sbrB}-P*sbrB*-P*lexA*	pTT_{con}-P*sbrB*-P*lexA* carrying a deletion of the *sbrB* TT	This study
pTT_{con}-P*sbrB*-P*fmtC*	pTT_{con}- P*sbrB*-P*lexA* carrying the P*fmtC* promoter instead of the LexA promoter	This study
pTT_{15981}-P*sbrB*-P*fmtC*	pTT_{con}- P*sbrB*-P*lexA* carrying the 15981 *sbrB* TT instead of the consensus *sbrB* TT and the P*fmtC* promoter instead of the LexA promoter	This study
pΔTT_{sbrB}-P*sbrB*-P*fmtC*	pTT_{con}- P*sbrB*-P*lexA* carrying a deletion of the *sbrB* TT and the P*fmtC* promoter instead of the LexA promoter	This study
pTT_{con}-P*blaZ*-P*lexA*	pTT_{con}- P*sbrB*-P*lexA* carrying P*blaZ* promoter instead of the *sbrB* promoter	This study
pTT_{15981}-P*blaZ*-P*lexA*	pTT_{con}- P*sbrB*-P*lexA* carrying the 15981 *sbrB* TT instead of the consensus *sbrB* TT and the P*blaZ* promoter instead of the *sbrB* promoter	This study
pΔTT_{sbrB}-pP*blaZ* P*lexA*	pTT_{con}- P*sbrB*-P*lexA* carrying the *sbrB* TT deletion and the P*blaZ* promoter instead of the *sbrB* promoter	This study
pΔP*sbrB*- TT_{15981}P*lexA*	pΔP*sbrB*- TT_{15981}P*lexA* carrying the P_{sbrB} promoter deletion and the 15981 *sbrB* TT instead of the consensus *sbrB* TT	This study

Continued in the following page

Table S2. *Continued*

Plasmids	Relevant characteristic(s)	Reference
pP*sbrB*-*che*-TT_{con}-*gfp*	pCN56 plasmid including a dual fluorescent reporter system monitoring transcriptional terminator read-through. The expression of mCherry and GFP indicates the levels of transcript upstream and downstream of selected TTs, respectively. The system is under the control of the P_{sbrB} promoter. This construct carries the consensus *sbrB* TT	This study
pP*sbrB*-*che*-TT_{15981}-*gfp*	pP*sbrB*-*che*-TT_{con}-*gfp* carrying the 15981 *sbrB* TT instead of the consensus *sbrB* TT	This study
pΔP*sbrB*-*che*-TT_{15981}-*gfp*	pP*sbrB*-*che*-TT_{con}-*gfp* carrying the *sbrB* promoter deletion and the 15981 sbrB TT instead of the consensus *sbrB* TT	This study
pP*sbrB*-*che*-$TT1828_{15981}$-*gfp*	pP*sbrB*-*che*-TT_{con}-*gfp* carrying TT1828 from *S. aureus* 15981 instead of the consensus *sbrB* TT	This study
pP*sbrB*-*che*-$TT1828_{res}$-*gfp*	pP*sbrB*-*che*-TT_{con}-*gfp* carrying the mispairing-restored TT1828 instead of the consensus *sbrB* TT	This study
pP*sbrB*-*che*-$TT1022_{con}$-*gfp*	pP*sbrB*-*che*-TT_{con}-*gfp* carrying consensus TT1022 instead of the consensus *sbrB* TT	This study
pP*sbrB*-*che*-$TT1022_{res}$-*gfp*	pP*sbrB*-*che*-TT_{con}-*gfp* carrying the mispairing-restored TT1022 instead of the consensus *sbrB* TT	This study
pP*sbrB*-*che*-$TT619_{con}$-*gfp*	pP*sbrB*-*che*-TT_{con}-*gfp* carrying consensus TT619 instead of the consensus *sbrB* TT	This study
pP*sbrB*-*che*-$TT619_{res}$-*gfp*	pP*sbrB*-*che*-TT_{con}-*gfp* carrying the mispairing-restored TT619 instead of the consensus *sbrB* TT	This study

Table S3. Oligonucleotides used in this study

Oligonucleotide name	Sequence [a]
**Construction of the pMAD plasmid for *sbrB* TT deletion**	
LB108	*CCCGGG*CGACGGTATTTTATGATAAGCATC
LB17	GTTACACAAATTAAAACCATTATTATTTTTTTCTTTCTTTTTTATTAAG
LB18	AAAAATAATAATGGTTTTAATTTGTGTAACATTTTGCAGAAATC
LB109	*GGATCC*CCAATTTACCTAGACAATGTTGC
**Construction of the pMAD plasmid for A112G mutation**	
LB108	*CCCGGG*CGACGGTATTTTATGATAAGCATC
LB109	*GGATCC*CCAATTTACCTAGACAATGTTGC
LB128	GGCTCGCTCCTGTAAATTATTACGGGGGCG
LB129	AATAATTTACAGGAGCGAGCCATTATTATTTTTTTC
**Construction of the pMAD plasmid for deletion of *sbrB* promoter**	
PSigB_LexA_A	*GGATCC*GTAGAGTGCGTTACGAACAA
PSigB_LexA_B	*GCGGCC*GCTTAACCAGTATAATTTTCTGTCC
PSigB_LexA_C_SacI	*GCCGGC*GACTTGAACGCGTCAAA
PSigB_LexA_D	*CCATGG*GTCAAACCATAGCAGAAAAT
PSigB_LexA_E	ATACAATACACCTAAATCGG
**Construction of the pMAD plasmid for substituting *sbrB* promoter by *blaZ* promoter**	
LB108	*CCCGGG*CGACGGTATTTTATGATAAGCATC
LB110	GATATTACAATTGTAATATTATGAATTCGTAAGGAAGTAGATAAACATGATGAC
LB111	ATAATATTACAATTGTAATATCGGTGTCAAT
LB109	*GGATCC*CCAATTTACCTAGACAATGTTGC
**Construction of pCN57-*sbrB* plasmid**	
LB35	*CTGCAG*TGTAAGGAAGTAGATAAACATGATGAC
LB12	*CCCGGG*ATATAAATTTTGAATTACAGATTTCTGC
**mRACE**	
LB75-OP-A	CTTGAACGCGTCAAAGATATAC
LB76-OP-B	ATATCCGCATCTTTTGGTTTATC
**Construction of pCN57$_{+1}$ plasmid**	
LB51	*GCATGC*AGCTTACTATGCCATTATTAATAACTTAG
LB106	A*CCCGGG*GATCCTCTAGAGTCGAC*GAATTC*ATAATATTACAATTGTAATATCGGTG TCAA
LB73	*GAATTC*GTCGACTCTAGAGGATCCCCGGGTACCGAGCTCCGTTAACTAATTAATT TAAGAAGGAGATATACATATG
LB74	AAGTGT*TGGCCATGG*AACAG
**Construction of pCN57$_{+1}$-*sbrB* plasmid**	
LB88	*GAATTC*TAAGGAAGTAGATAAACATGATGACAAC
LB12	*CCCGGG*ATATAAATTTTGAATTACAGATTTCTGC
**Construction of pPsbrB and pPsbrB RT plasmids**	
SalI_sigB_prom	*GTCGAC*AATTATACTGGTTAATGTTTTTGGCATGAGATTAAAGGGTAATGTTTGTC ATAAAGCAAGCATATAATAT
BamHI_GFP_end	*GGATCC*TTATTTGTATAGTTCATCCATGCCAT
LB1	*GCATGC*AATTATACTGGTTAATGTTTTTGGCATG
LB12	*CCCGGG*ATATAAATTTTGAATTACAGATTTCTGC

Continued in the following page

Table S3. *Continued*

Oligonucleotide name	Sequence [a]
**Construction of pTL plasmids carrying *sbrB* and its mutants**	
LB51	*GCATGC*AGCTTACTATGCCATTATTAATAACTTAG
LB2	*ACTAGT*TTTTTTTCTTTCTTTTTTATTAAGTATATCTTTGAC
LB102	*GAATTC*GTAACCAAGTAGATAAACATGATGACAACAG
LB118	*ACTAGT*CATCTTGTTTATCTACTTCCTTAC
LB119	*ACTAGT*CATCTTGTTTATCTACTTCCTTAC
LB6	CTGTTGTCTTCTTGTTTATCTACTTCCTTACAGAC
LB5	AGTAGATAAACAAGAAGACAACAGATAAACCAAAAG
LB41	CATGATGACAACAGATTAACCAAAAGATGCGGATAT
LB42	CTTTTGGTTAATCTGTTGTCATCATGTTTATCTACTT
LB56	CGCGTCAAAGATATACTTAATAAAAAAG
LB74	AAGTGTTGGCCATGGAACAG
LB11	TTCTTTCTTTTTTATTAAGTATATCTTTGACGCGTTAAAGTATATCCGCATCTTTTGG
**Construction of the pRT plasmids carrying *sbrB* and its mutants**	
LB101	*GAATTC*GTAAGGAAGTAGATAAACATGATGACAAC
LB12	*CCCGGG*ATATAAATTTTGAATTACAGATTTCTGC
LB102	*GAATTC*GTAACCAAGTAGATAAACATGATGACAACAG
LB112	*GAATTC*GTAAGGAAGTAGATAAACAAGATGACAACAG
LB113	*GAATTC*GTAAGGAAGTAGATAAACAAGAAGACAACAGATAAAC
pCN_Univ_fw_AT	*CCGTATTA*CCGCCTTTGAGTG
LB62	AAACGTGCTTATATTTTTATCGATAATACAACCATTATTATTTTTTTCTTTCTTTTTTATTAAGTATATC
LB61	TTGTATTATCGATAAAAATATAAGCACGTTTCTCACTCCTGTAAATTATTACGG
LB17	GTTACACAAATTAAAACCATTATTATTTTTTTCTTTCTTTTTTATTAAG
LB18	AAAAATAATAATGGTTTTAATTTGTGTAACATTTTGCAGAAATC
**Construction of the pRT plasmids carrying *sbrB* TT and its variants**	
LB101	*GAATTC*GTAAGGAAGTAGATAAACATGATGACAAC
LB12	*CCCGGG*ATATAAATTTTGAATTACAGATTTCTGC
LB128	GGCTCGCTCCTGTAAATTATTACGGGGGCG
LB129	AATAATTTACAGGAGCGAGCCATTATTATTTTTTTC
LB124	CCGTAATAATTTACAAGAGCGAGCCATTATTATTTTT
LB125	GCTCTTGTAAATTATTACGGGGGCGAGTTTTAATTTGTGT
LB196	TCGCCTCCGTAATAATTTACAGGAG
LB197	GTAAATTATTACGGAGGCGAGTTTTAATTTGTGTAAC
LB194	CCCCGTAATAATTTACAGGAGC
LB195	GCTCCTGTAAATTATTACGGGGCGAGTTTTAATTTGTGTAACATTTTG
LB122	CCCCCCGTAATAATTTACAGGAG
LB123	GTAAATTATTACGGGGGGCGAGTTTTAATTTG
LB192	TGCCCCCGTAATAATTTACAAGAGCGAGCCATTATTATTTTTTTC
LB193	TTGTAAATTATTACGGGGGCAAGTTTTAATTTGTGTAACATTTTGC
LB198	CACAAATTAAAATTCGCCCCCGTAATAATTTA
LB199	GGGGCGAATTTTAATTTGTGTAACATTTTGCAG
LB66	GATCCCTCAATCGAGGGGTCTTTTTTTAATTTGTGTAACATTTTGCAGAAAT
LB67	AAAAAAAGACCCCTCGATTGAGGGATCCCATTATTATTTTTTTCTTTCTTTTTTATTAAGTATATCT
**Construction of the pRT plasmids carrying *sbrB* from different *Staphylococcus* species**	
LB136	*GAATTC*GTAA*GGATG*TAGATAAATATGAAGCAAAAA
LB137	*CCCGGG*TTTAAGTGTAGGCCTCCTTATATTTT
LB138	*GAATTC*GTAA*GGATG*TAGATAAGCATGAAAAC
LB139	*CCCGGG*AGCCCTCCTATATCAAGAC
LB140	*GAATTC*GTAAGGAAGTAGATAAACATGATGAT
LB141	*CCCGGG*TGTTACAACACTAGAAACAATCAGA

Continued in the following page

Table S3. *Continued*

Oligonucleotide name	Sequence [a]
**Construction of the pCN56 reporter plasmids**	
LB114	*CTCGGTACC*TTAACGAACAAATGTTTGTTTTGGTTTCAAATTAATGATAT ACTGTATTTAGTAGGGGGTTATAAAAAATGACTAG
LB93	*GGATCC*TTATTTGTATAATTCATCCATACCACCAG
LB1	*GCATGC*AATTATACTGGTTAATGTTTTTGGCATG
LB92	*GGATCC*TATTTTAAACCGTTATATATTATCGTAATTGTTAAG
LB154	TTATCTTCTTCACCTTTACTAGTCATTTTTTATAACCCCCTACTTTATTTCT GTTATAAATCAAAATATATCATATAAAC
LB155	GTTAACGGAGCTCGGTACCGTTATATAAATCAAAGGTAAATGATATGCT ATTTTTATGGTTTATATGATATATTTTGATTTATAACAG
LB129	AATAATTTACAGGAGCGAGCCATTATTATTTTTTTC
LB128	GGCTCGCTCCTGTAAATTATTACGGGGGCG
LB17	GTTACACAAATTAAAACCATTATTATTTTTTTCTTTCTTTTTTATTAAG
LB18	AAAAATAATAATGGTTTTAATTTGTGTAACATTTTGCAGAAATC
LB128	GGCTCGCTCCTGTAAATTATTACGGGGGCG
**Construction of the pCN56 reporter plasmid expressing the *sbrB* terminators**	
LB64	*GCATGC*TTTGTCTGTAAGGAAGTAGATAAACATG
LB92	*GGATCC*TATTTTAAACCGTTATATATTATCGTAATTGTTAAG
LB159	*GGATCC*GTAAGGAAGTAGATAAACATGATGACA
LB160	ATTTAGAATAG*GCGCGCC*TTA
LB190	AATGCCTA*GGATCC*TAATGGCTCGCTCCTGTAAATTATTACGGGGGCGA GTTTTAATTTGTGTAACATTTTGCAG
LB189	TAACGGAGCTCGGTA*CCCGGG*ATATAAATTTTGAATTACAGATTTCTGC AAAATGTTACACAAATT
TERM1828_BamHI_fw	*GGATCC*AATAATATAAGTATGACTAAAGCCAC
TERM1828_SmaI_rev	*CCCGGG*CCCTAACTCCCCACAAAT
TERM1828_con_fw	*GGATCC*AATAATATAAGTATGACTAAAGCCACATCCAATATAGGACGTG GCTTTT
TERM1828_res_fw	*GGATCC*AATAATATAAGTATGACTAAAGCCACGTCCAATATAGGACGTG GCTTTT
TERM1022_rev	GCCAAGCTAAAGGTAAAGG
TERM1022_KpnI	GGTACCCGGACCACACCTCTAAAAAAAGC
TERM1022_res_fw	*GGTAC*CCGGACCACACCTCTAAAAAAAGCGTAGGTTAATTTAACCT
TERM619_BamHI	*GGATCC*GAAGATATCTTCCGGTAAAGTGG
TERM619_SmaI	*CCCGGG*CCTTTAAATAAATAAGTTAAGTACAAAC
TERM619_res	*GGATCC*GAAGATATCTTCCGGTAAAGTGGGCAATTTAAATTGCTTAGTG AGACCTATGCTATTTAT
**Construction of probes for the Northern blot assays**	
LB77	TTACATTTCGCGGTACAAAC
LB78	TAATACGACTCACTATAGGGTTGTAAGTGATCAAACAAATG
LB79	TTGTAAGTGATCAAACAAATG
LB80	TAATACGACTCACTATAGGGTTACATTTCGCGGTACAAAC
sosA fw1	CTAGGAGTGAAAATGATG
sosA T7	TAATACGACTCACTATAGGGGACAATGTATCAATTTATTAAAGC
sRNA antilexA T7	AATACGACTCACTATAGGGAAATTAAAACTCGCCCCCG
sRNA antilexA fw2	TGAACGCGTCAAAGATATACTT

[a]*Restriction enzymes are indicated in italic*

Table S4. Summary of TT$_{sbrB}$ variants found in *S. aureus* strains

Variant	Mutation	Consequence	Representative Strain	Accession number	Number of strains	Free energy ΔG	SbrB TT sequence<br>Left arm Loop Right arm Poly U
Con	-	-	MW2		9881	-22.5	GGCUCGCUCCUGUAAAUUAUUACGGGGGCGAGUUUUAAUUU
V1	G112A	Mispairing	15981	-	1	-17.1	GGCUC**A**CUCCUGUAAAUUAUUACGGGGGCGAGUUUUAAUUU
V2	C116U	Wobble pair	MN8	CM000952.1	478	-19.7	GGCUCGCUC**U**UGUAAAUUAUUACGGGGGCGAGUUUUAAUUU
V3	G132A	Mispairing	3688STDY6124954	FQJV01000002.1	1	-16.5	GGCUCGCUCCUGUAAAUUAUUACGG**A**GGCGAGUUUUAAUUU
V4	G133Δ	Mispairing	RF122	CAACVQ010000005.1	6	-18.4	GGCUCGCUCCUGUAAAUUAUUACGGGG**-**CGAGUUUUAAUUU
V5	G134::G	Mispairing	O267	CP034102.1	5	-19.8	GGCUCGCUCCUGUAAAUUAUUACGGGGG**G**CGAGUUUUAAUUU
V6	C116U/G136A	Wobble pair / Mispairing	MRSA	FKXV01000017.1	1	-13.7	GGCUCGCUC**U**UGUAAAUUAUUACGGGGGC**A**AGUUUUAAUUU
V7	G138A	Mispairing	AL-699	MOMP01000002.1	1	-18.2	GGCUCGCUCCUGUAAAUUAUUACGGGGGCGA**A**UUUUAAUUU
V8	G136A	Mispairing	ST1464	ANIT01000056.1	2	-16.5	GGCUCGCUCCUGUAAAUUAUUACGGGGGC**A**AGUUUUAAUUU
V9	C135U	Wobble pair	M6K146	BECE01000002.1	1	-20.6	GGCUCGCUCCUGUAAAUUAUUACGGGGG**U**GAGUUUUAAUUU
V10	C116U/C135U	Wobble pair / Wobble pair	st1643	FGNX01000021.1	1	-17.8	GGCUCGCUC**U**UGUAAAUUAUUACGGGGG**U**GAGUUUUAAUUU
V11	G133A	Mispairing	CM47	PZXG01000015.1	1	-23.4	GGCUCGCUCCUGUAAAUUAUUACGGG**A**GCGAGUUUUAAUUU
V12	C113U	Wobble pair	NA	FKLX01000001.1	1	-20.0	GGCUCG**U**UCCUGUAAAUUAUUACGGGGGCGAGUUUUAAUUU
V13	U139G	Mispairing	1801-1 2010	JOPS01000022.1	9	-21.2	GGCUCGCUCCUGUAAAUUAUUACGGGGGCGAG**G**UUUAAUUU
V14	G131U	Mispairing	USFL091	CHEO01000001.1	1	-15.9	GGCUCGCUCCUGUAAAUUAUUACG**U**GGGCGAGUUUUAAUUU
V15	A137G	Wobble pair	Lyso 2 2010	JOPN01000025.1	1	-21.6	GGCUCGCUCCUGUAAAUUAUUACGGGGGCG**G**GUUUUAAUUU
V16	A120G	Wobble pair	364P	PDIS01000001.1	2	-22.6	GGCUCGCUCCUGU**G**AAUUAUUACGGGGGCGAGUUUUAAUUU
V17	U139A	Mispairing	SA-085	JXIF01000131.1	1	-21.1	GGCUCGCUCCUGUAAAUUAUUACGGGGGCGAG**A**UUUAAUUU
V18	U123C	Loop	PN246B0	PDVB01000142.1	1	-22.5	GGCUCGCUCCUGUAAA**C**UAUUACGGGGGCGAGUUUUAAUUU
V19	C115A	Mispairing	CM184	PZTA01000041.1	1	-16.5	GGCUCGCU**A**CUGUAAAUUAUUACGGGGGCGAGUUUUAAUUU
V20	G130A	WC pair	M6K137	BEBZ01000007.1	1	-23.2	GGCUCGCUCCUGUAAAUUAUUAC**A**GGGGCGAGUUUUAAUUU
V21	G138C	Mispairing	st1854	FGTH01000001.1	1	-17.9	GGCUCGCUCCUGUAAAUUAUUACGGGGGCGA**C**UUUUAAUUU
V22	U119C	Mispairing	GGMC6008	JBOF01000002.1	1	-18.0	GGCUCGCUCCUG**C**AAAUUAUUACGGGGGCGAGUUUUAAUUU

Table S5. Supplementary excel file including the results of the genome-wide transcriptional terminator prediction and transcriptional read-through analysis in *Staphylococcus aureus*. *In silico* predictions of intrinsic Rho-independent transcriptional terminators were performed by the TransTermHP v2.07 program as previously described (7) using the *S. aureus* NCTC 8325 genome (NC_007795.1) as a reference. The ∆G of predicted TTs was calculated using the Quickfold program (8). The level of the transcriptional read-through for each predicted TT, was calculated using our previous RNA sequencing data from the *S. aureus* 15981 strain (9). Predicted TTs are ordered according their read-through levels. Predicted TTs that were not included in the read-through ratio calculation are shown in grey characters (genes with transcription levels below 4).

SUPPLEMENTARY FIGURES

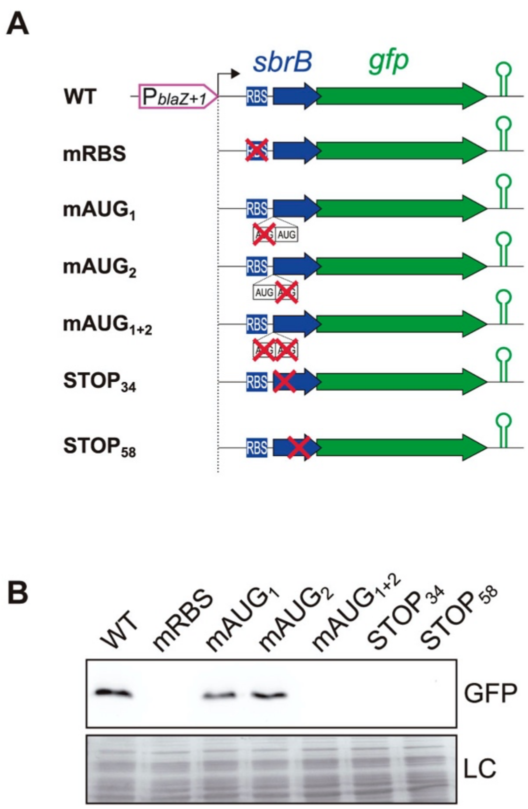

Figure S1. Translational reporters showing the effect of *sbrB* mRNA mutations. (**A**) Schematic representation of the different plasmids harbouring *sbrB* translation reporters. WT and mutant mRNAs were expressed under the control of the P_{blaZ+1} promoter. (**B**) Western blot analyses showing the GFP levels produced from the different plasmids. Membranes were incubated with monoclonal anti-GFP and developed with peroxidase-conjugated goat anti-mouse antibodies and a bioluminescence kit. Coomassie gel portions are included as loading controls (LC).

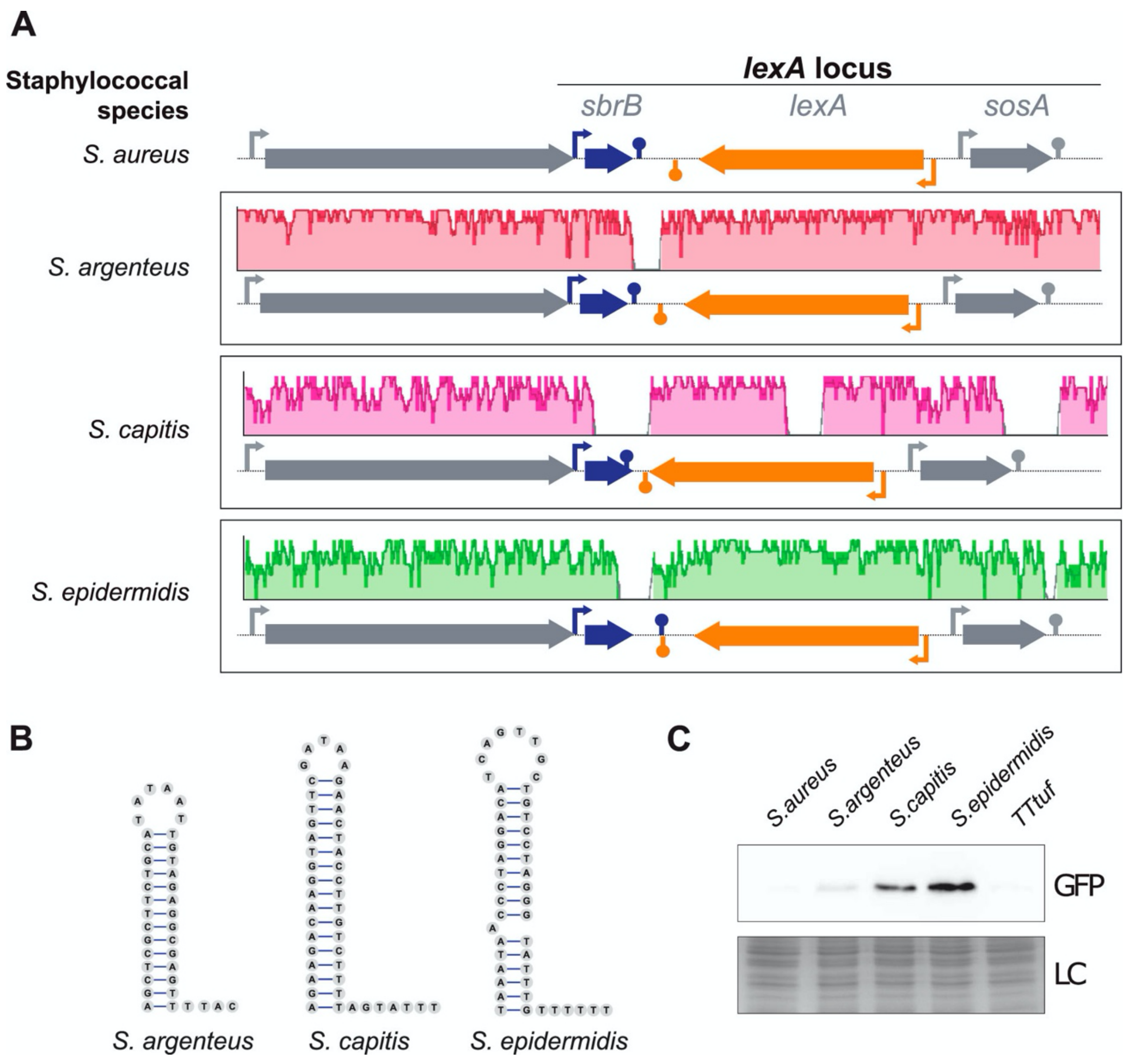

Figure S2. TT_{sbrB} is not conserved among staphylococcus species. (A) Genomic comparison of the *sbrB*-*lexA*-*sosA* locus using Mauve, a multiple genome alignment tool (10). The conserved DNA regions when *S. aureus* is compared with *S. argenteus*, *S. capitis* and *S. epidermidis* are indicated in red, pink and green, respectively.(**B**) Transcriptional terminator structures of the *sbrB* mRNA from *S. argenteus*, *S. capitis* and *S. epidermidis* were predicted using RNAstructure (11). (**C**) Western Blot analysis showing the TT_{sbrB} read-through levels. The *sbrB* mRNA from different staphylococcal species was cloned into the pRT-*gfp* plasmid and GFP levels were developed using monoclonal anti-GFP antibodies.

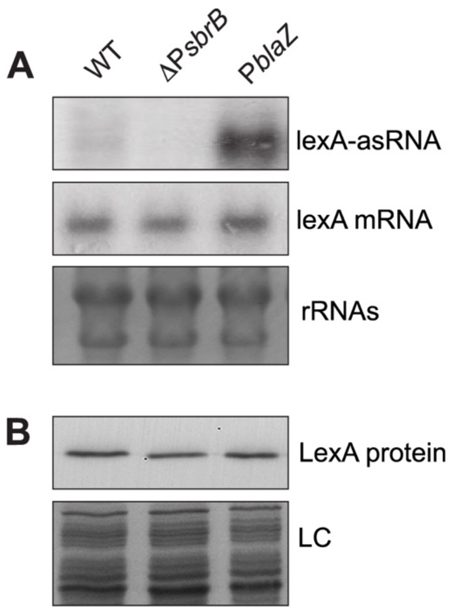

Figure S3. Variations in the chromosomic *lexA*-asRNA levels do not influence LexA expression. (**A**) Northern blot analysis showing the *lexA*-asRNA and *lexA* mRNA levels in the WT, Δ*PsbrB* and P*blaZ* strains. The indicated transcript levels were developed using specific ^{32}P-radioactive labelled riboprobes. Ribosomal rRNAs stained with Midori Green were included as loading controls. (**B**) Western blot analysis showing the levels of the LexA protein expressed from the same strains, which were detected by using specific anti-LexA antibodies. Coomassie gel portions are included as loading controls (LC).

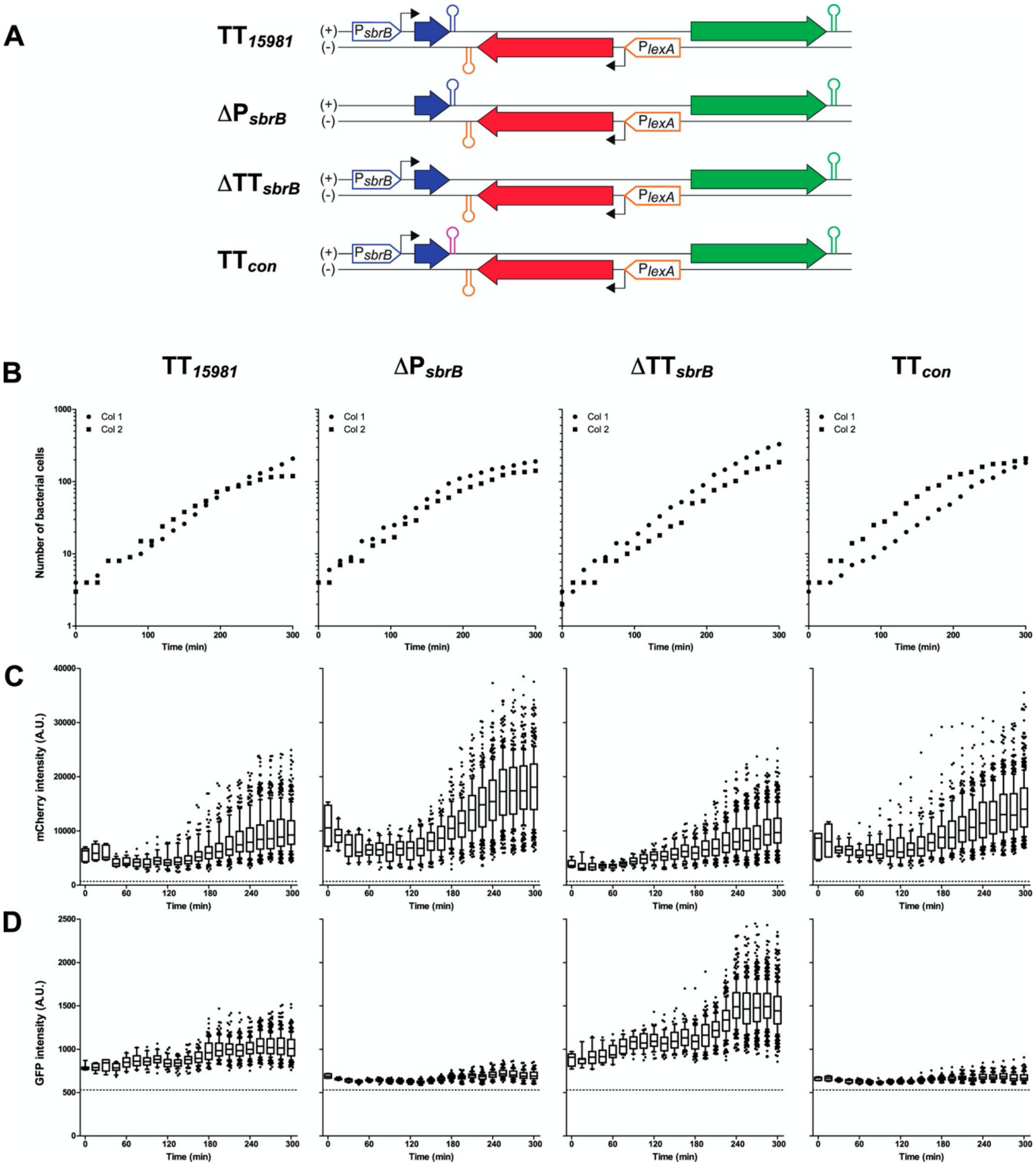

Figure S4. Variations of *lexA*-asRNA levels affect the LexA reporter expression in *S. aureus* subpopulations. (A) Schematic representation of dual fluorescent reporter plasmids used to monitor single cell expression in time-lapse fluorescence microscopy. (**B**) Number of bacteria per microcolony counted every 15 min. (**C**) Box plots showing the mCherry levels in each bacterial cell (**D**) Box plots showing the GFP levels in each bacterial cell. The strains transformed with the reporter plasmids carrying TT_{15981}, ΔP_{sbrB}, ΔTT_{sbrB} and the TT_{con} (TT from the MW2) variants were grown at 37ºC in CellAsic microfluidic plates with a continuous flow of MHg and challenged with 30 mM of KOH during 4 hours. Images were taken in intervals of 15 min. mCherry and GFP intensities in single cells were quantified using the ROI statistics plugin of the Icy biomage software (12) (http://icy.bioimageanalysis.org).

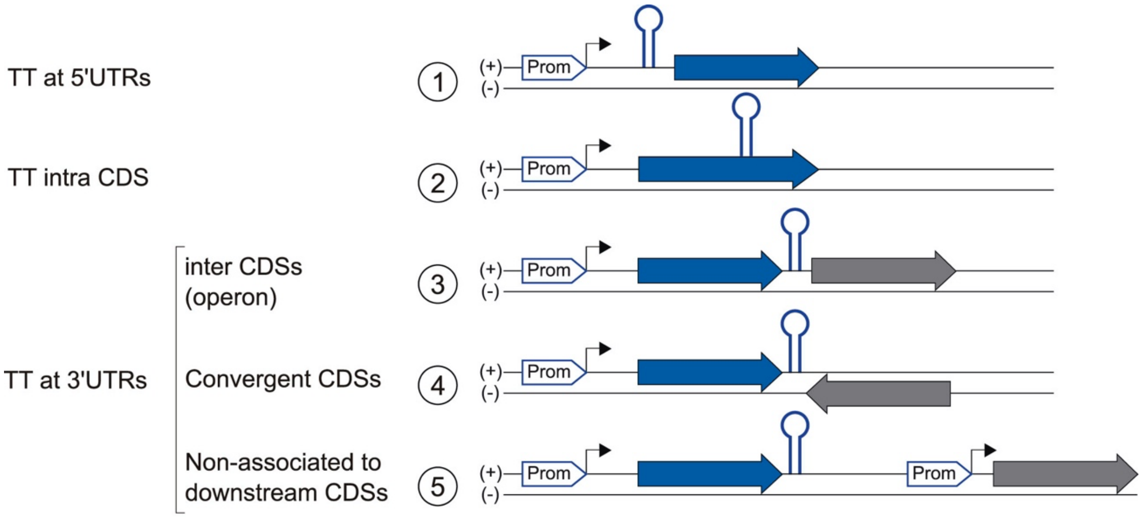

Figure S5. Classification of predicted TTs according to their position relative to their corresponding CDS. Predicted TTs could be located at the 5'UTRs (1), into a CDS region (2), between two CDSs of an operon (3), between convergent genes (4) and downstream of their corresponding CDSs without any association to downstream genes (5).

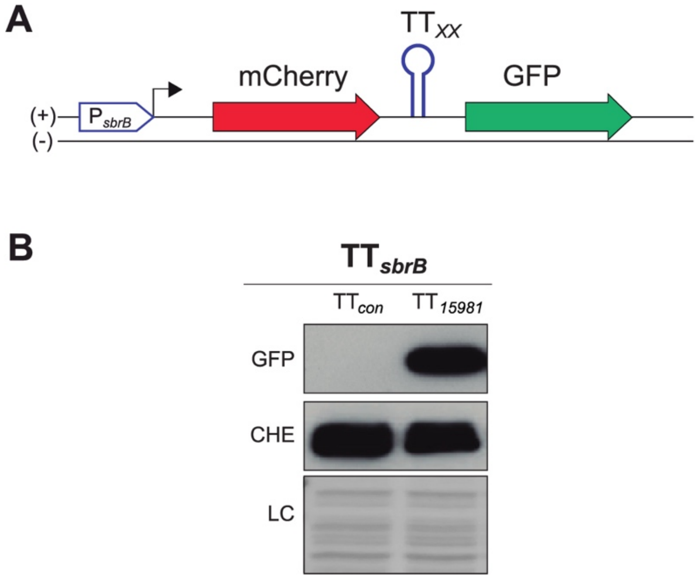

Figure S6. Design of a dual fluorescent reporter plasmid for monitoring TREs. (A) Schematic representation of the dual fluorescent reporter plasmid designed to monitor transcriptional terminator efficiencies. The expression of chimeric transcripts is controlled by the P$_{sbrB}$ promoter. (**B**) As controls, the transcriptional terminator efficiencies of the TT$_{sbrB}$ from *S. aureus* 15981 (TT$_{15981}$) and MW2 (TT$_{con}$) strains were monitored by Western blots as indicated in Figure 6. A coomassie stained gel portion is included as loading control.

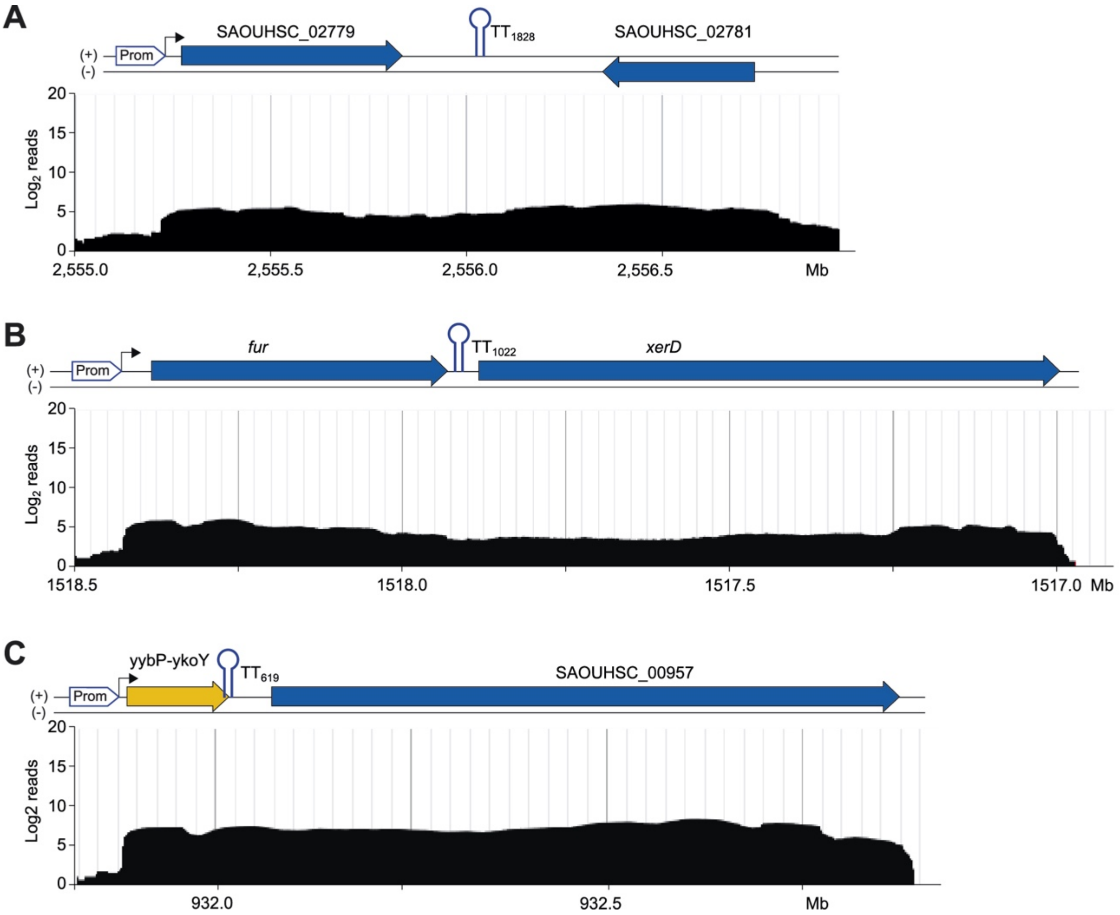

Figure S7. Transcriptomic map of genomic regions included predicted TTs producing TREs. Jbrowser images showing the transcript levels from SAOUHSC_02779 (**A**), *fur* (**B**) and *yybP-ykoY* (**C**) chromosomic regions in the *S. aureus* 15981. The complete transcriptomic maps are available at http://rnamaps.unavarra.es (9).

SUPPLEMENTARY REFERENCES

1. Valle,J., Toledo-Arana,A., Berasain,C., Ghigo,J.-M., Amorena,B., Penadés,J.R. and Lasa,I. (2003) SarA and not sigma B is essential for biofilm development by *Staphylococcus aureus*. *Mol Microbiol*, **48**, 1075–1087.

2. Baba,T., Takeuchi,F., Kuroda,M., Yuzawa,H., Aoki,K.-I., Oguchi,A., Nagai,Y., Iwama,N., Asano,K., Naimi,T., *et al.* (2002) Genome and virulence determinants of high virulence community-acquired MRSA. *Lancet*, **359**, 1819–1827.

3. Arnaud,M., Chastanet,A. and Debarbouille,M. (2004) New vector for efficient allelic replacement in naturally nontransformable, low-GC-content, Gram-positive bacteria. *Appl Environ Microbiol*, **70**, 6887–6891.

4. Charpentier,E., Anton,A.I., Barry,P., Alfonso,B., Fang,Y. and Novick,R.P. (2004) Novel cassette-based shuttle vector system for gram-positive bacteria. *Appl Environ Microbiol*, **70**, 6076–6085.

5. Catalan-Moreno,A., Cela,M., Menendez-Gil,P., Irurzun,N., Caballero,C.J., Caldelari,I. and Toledo-Arana,A. (2021) RNA thermoswitches modulate *Staphylococcus aureus* adaptation to ambient temperatures. *Nucleic Acids Res*, **49**, 3409–3426.

6. Balestrino,D., Hamon,M.A., Dortet,L., Nahori,M.-A., Pizarro-Cerda,J., Alignani,D., Dussurget,O., Cossart,P. and Toledo-Arana,A. (2010) Single-cell techniques using chromosomally tagged fluorescent bacteria to study *Listeria monocytogenes* infection processes. *Appl Environ Microbiol*, **76**, 3625–3636.

7. Kingsford,C.L., Ayanbule,K. and Salzberg,S.L. (2007) Rapid, accurate, computational discovery of Rho-independent transcription terminators illuminates their relationship to DNA uptake. *Genome Biol*, **8**, R22.

8. Zuker,M. (2003) Mfold web server for nucleic acid folding and hybridization prediction. *Nucleic Acids Res*, **31**, 3406–3415.

9. Lasa,I., Toledo-Arana,A., Dobin,A., Villanueva,M., de los Mozos,I.R., Vergara-Irigaray,M., Segura,V., Fagegaltier,D., Penadés,J.R., Valle,J., *et al.* (2011) Genome-wide antisense transcription drives mRNA processing in bacteria. *Proc Natl Acad Sci USA*, **108**, 20172–20177.

10. Darling,A.C.E., Mau,B., Blattner,F.R. and Perna,N.T. (2004) Mauve: multiple alignment of conserved genomic sequence with rearrangements. *Genome Res*, **14**, 1394–1403.

11. Reuter,J.S. and Mathews,D.H. (2010) RNAstructure: software for RNA secondary structure prediction and analysis. *BMC Bioinformatics*, **11**, 129–9.

12. de Chaumont,F., Dallongeville,S., Chenouard,N., Hervé,N., Pop,S., Provoost,T., Meas-Yedid,V., Pankajakshan,P., Lecomte,T., Le Montagner,Y., *et al.* (2012) Icy: an open bioimage informatics platform for extended reproducible research. *Nat Meth*, **9**, 690–696.
